# Insights into the biodegradation of polycaprolactone through genomic analysis of two plastic-degrading *Rhodococcus* bacteria

**DOI:** 10.3389/fmicb.2023.1284956

**Published:** 2024-01-03

**Authors:** Jessica Zampolli, Daniele Vezzini, Stefania Brocca, Patrizia Di Gennaro

**Affiliations:** Department of Biotechnology and Biosciences, University of Milano-Bicocca, Milan, Italy

**Keywords:** biodegradable plastic, *Rhodococcus opacus*, *Rhodococcus erythropolis*, polycaprolactone, aliphatic polyester, hydrolytic activity, gene clustering

## Abstract

Polycaprolactone (PCL) is an aliphatic polyester often utilized as a model to investigate the biodegradation potential of bacteria and the involved catabolic enzymes. This study aims to characterize PCL biodegradative metabolic potential and correlate it to genomic traits of two plastic-degrading bacteria—*Rhodococcus erythropolis* D4 strain, a new isolate from plastic-rich organic waste treatment plant, and *Rhodococcus opacus* R7, known for its relevant biodegradative potential on polyethylene and similar compounds. After preliminary screening for bacteria capable of hydrolyzing tributyrin and PCL, the biodegradation of PCL was evaluated in *R*. *erythropolis* D4 and *R*. *opacus* R7 by measuring their growth and the release of PCL catabolism products up to 42 days. After 7 days, an increase of at least one order of magnitude of cell number was observed. GC-MS analyses of 28-day culture supernatants showed an increase in carboxylic acids in both *Rhodococcus* cultures. Furthermore, hydrolytic activity (~5 U mg^−1^) on short/medium-chain *p*-nitrophenyl esters was detected in their supernatant. Finally, a comparative genome analysis was performed between two *Rhodococcus* strains. A comparison with genes annotated in reference strains revealed hundreds of gene products putatively related to polyester biodegradation. Based on additional predictive analysis of gene products, gene expression was performed on a smaller group of genes, revealing that exposure to PCL elicits the greatest increase in transcription for a single gene in strain R7 and two genes, including that encoding a putative lipase, in strain D4. This work exhibits a multifaceted experimental approach to exploit the broad potential of *Rhodococcus* strains in the field of plastic biodegradation.

## 1 Introduction

The use of plastics is widespread because of their attractive features, as well as their well-established production technology and affordable price. Indeed, global plastic production reached approximately 391 million tons in 2021 (Plastics Europe, 2022). The consequent waste production and the ubiquitous spread of plastic are causing environmental pollution and energy crises that have reached alarming levels. In order to support and address these issues, the production of degradable aliphatic polyesters has been promoted (Mo et al., 2023). Polycaprolactone (PCL), among others, such as polyethylene succinate, polylactic acid, polyhexylene succinate, polybutylene succinate, and polyethylene furan acetate, represents a model for studying polyester biodegradability and the phenotypical and molecular features of microorganisms that catabolize biodegradable aliphatic polyesters (Ma et al., 2020; Kosiorowsk et al., 2021). PCL is a linear polyester composed of 6-hydroxyhexanoic acid, chemically synthesized by ring-opening polymerization of ε-caprolactone. The resulting polymer is semi-crystalline; its crystallinity tends to decrease with increasing molecular weight. Its low melting point (59–64°C), good solubility, and blend compatibility make PCL useful as an additive, ceramic or metal binder, adhesive, and ink component in both biomedical and agricultural fields (Woodruff and Hutmacher, 2010; Mo et al., 2023).

In general, the biodegradation of plastics by microorganisms is considered an eco-friendly solution for plastic waste disposal. This is due to both abiotic hydrolysis and microbial ability to hydrolyze the main chain of polymers and consequently internalize and catabolize the generated smaller polymer molecules (as carboxylic acids for PCL, Hakkarainen, 2003), converting them into inorganic compounds, i.e., CO_2_. However, the rate of the biodegradation process determines whether the polymer is biodegradable (Suzuki et al., 2021). For example, the European standard EN 13432, which defines the requirements for compostable plastic packaging, states that its biodegradability should consist of the loss of 90% of mass within 6 months.

The microbial biodegradability of PCL has been observed in diverse environments, probably due to its similarity to the natural polymeric compound cutin (Suzuki et al., 2021). Multiple studies have reported both aerobic and anaerobic PCL-degrading bacteria or fungi, including *Acinetobacter* spp., *Alcaligenes faecalis, Bacillus* spp., *Brevundimonas* sp., *Clostridium* sp., *Lactobacillus* spp., *Pseudomonas* spp., *Streptomyces* spp., *Aspergillus* spp., *Fusarium* spp., *Mucor* spp., *Paecilomyces lilacinus, Penicillium* spp., and *Rhizopus* spp. (Murphy et al., 1996; Tiago et al., 2004; Tokiwa et al., 2009; Nawaz et al., 2014; Khan et al., 2017; Bartnikowski et al., 2019; Mandic et al., 2019; Suzuki et al., 2021; Budkum et al., 2022; Atanasova et al., 2023). The degradation of PCL is carried out by esterases and/or depolymerases secreted extracellularly. Among them, the most studied enzymes belong to the classes of carboxylic-ester hydrolases (EC 3.1.1), and more particularly lipases (EC 3.1.1.3) and cutinases (EC 3.1.1.74). This activity is usually detected by hydrolysis assay of *p*-nitrophenyl esters with chains of various lengths (C2–C18) (Tsuboi et al., 2022; Won et al., 2022). However, it is difficult to classify these enzymes on the basis of substrate specificity alone (Suzuki et al., 2021).

Among contaminant-degrading microorganisms, bacteria of the *Rhodococcus* genus are characterized by remarkable physiological and functional features, often associated with unique and diversified enzymatic capabilities demonstrating their environmental and biotechnological importance (Alvarez, 2019). Rhodococci are widespread in several environments and show a broad spectrum of metabolic capabilities and an intriguing potential for plastic degradation (Zampolli et al., 2019; Li et al., 2022). However, only two studies have reported the ability of bacteria belonging to the *Rhodococcus* genus to degrade PCL (Urbanek et al., 2017; Won et al., 2022; Atanasova et al., 2023). A cutinase (Rcut) was isolated from one of these strains, showing high activity on medium-length (C4–C10) fatty acid esters, although cutinases normally prefer substrates with short-chain substrates (Won et al., 2022).

Currently, the advent of high-throughput-omics technologies provides access to the analysis of entire biological systems, with the additional possibility of predicting and constructing catabolic pathways (Kobras et al., 2021). In the present scenario, the characterization of biodegradable polyester-degrading bacteria and their hydrolytic functions appear more accessible for researchers; thus, a widespread contribution to the knowledge of plastic degradation processes is to be expected.

The aim of the present study is to characterize the PCL biodegradative ability of two *Rhodococcus* bacteria—*Rhodococcus erythropolis* D4 and *Rhodococcus opacus* R7, to provide, for the first time, a correlation between genetic and functional traits.

*Rhodococcus erythropolis* D4 has been isolated from a plastic-rich organic waste treatment plant, and its genome has been completely sequenced. *R*. *opacus* R7 has been extensively evaluated for its ability to degrade a broad range of contaminants, including aliphatic, mono- and polycyclic hydrocarbons, naphthenic acids, and polyethylene-based plastic, and its genome has shown peculiar and meaningful features (Di Gennaro et al., 2001; Orro et al., 2015; Zampolli et al., 2020, 2021). The R7 strain was selected for its diversified metabolic versatility to evaluate its metabolic potential toward PCL and investigate its genomic features with respect to *R*. *erythropolis* D4. Chosen for their hydrolytic activity on polyesters, both *Rhodococcus* strains were assayed for their ability to grow on PCL as the sole carbon and energy source and biodegrade PCL into metabolic products, e.g., carboxylic acids. The capability of *Rhodococcus* strains to hydrolyze aliphatic polyesters is likely due to the PCL-induced secretion of hydrolytic enzymes, which have been shown to be active on various *p*-nitrophenyl esters (*p*NP-esters). Finally, their genome analysis through annotation search, gene clustering, and whole-genome alignment against reference gene products provided a few gene products putatively related to polyester biodegradation for *R*. *erythropolis* D4 and *R*. *opacus* R7, validated by gene expression analysis.

## 2 Materials and methods

### 2.1 Bacterial strains and growth conditions

The microbial strains used in this study are *R. erythropolis* D4 (identification number 8B-C2-LD, deposited in the Private Collection of Microbiology Laboratory, BtBs Department of University of Milano-Bicocca), a new microbial isolate from a plastic and organic waste treatment plant, and *R. opacus* R7 (CIP identification number 107348, deposited in the Institute Pasteur Collection) (Di Gennaro et al., 2001). The two strains were routinely cultivated in Luria-Bertani (LB) medium and pre-cultured in M9 mineral medium (Maniatis et al., 1982) added with 20 mM malate (M9-M) and incubated overnight after the cells were washed in M9 mineral medium.

*Rhodococcus* cells pre-cultured in M9-M were washed in an M9 medium and resuspended in a fresh M9 medium to be inoculated in the presence of 1% *w/v* PCL (M9-PCL) as the sole carbon and energy source. *Rhodococcus* cultures in M9-PCL were established by adding commercial PCL powder (cod. 440752, average Mw ~1,400, average Mn ~10,000 by GPC, Merck, Italy) to each sterile flask. It was then dissolved in dichloromethane (DCM), evaporated overnight, and the M9 medium was added. Each *Rhodococcus* culture was inoculated from the corresponding pre-culture to obtain an initial optical density read at 600 nm (OD_600_) of 0.1. All cultures were incubated at 30°C under shaking (120 rpm).

### 2.2 Bacterial strain isolation and screening for polyester hydrolysis

A sludgy waste with plastic debris and microplastics was sampled from the final treatment step biowaste treatment plant in Lombardy, Italy (sampling date: 28 May 2021).

The total heterotrophic bacteria were evaluated using the colony-forming unit (CFU) count method by resuspending 1 g (~2 ml) of the sampled sludge in 9 ml M9 medium after serially diluting and plating on LB agar medium. The plates were incubated at 30°C for 48 h.

The bacteria isolation procedure was conducted by enrichment cultures established in 100-ml flasks containing 20 ml M9 medium and 1 g of the sampled sludgy waste (1:10 waste:medium volume ratio) and incubated at 30°C for 7 days. The same procedure was repeated three times under the same conditions. The enrichment culture was then serially diluted to 10^5^ before plating on LB agar plates to isolate individual bacterial colonies.

Each isolate was screened in a solid medium for its hydrolytic activity of PCL and tributyrin, a reference substrate for the detection of lipase/esterase activity. In particular, screening on commercial PCL or tributyrin (Merck, Italy) was accomplished by emulsifying each substrate with Ultra-Turrax (VWR International, Milano, Italy) in LB agar medium before its sterilization and distribution in Petri dishes (Molitor et al., 2020). 3-μl aliquots of the bacterial cultures of each isolate grown overnight on LB were spotted on the agar plates of emulsified substrates and incubated at 30°C for 48 h. The plates were then incubated at 4°C for 1 week to check for the presence of hydrolysis zones (i.e., clear halos on an opaque background around the cell spots) and evaluate the increase in their diameter. In addition, the screening of lipase and esterase activity was performed on *R*. *opacus* R7 to evaluate its efficiency, while *Escherichia coli* ATCC 25922 and Lipozyme CALB L (Merck, Italy) were used as negative and positive controls, respectively. *Escherichia coli* ATCC 25922 (American Type Culture Collection, Manassas, USA) was pre-cultured in LB medium and incubated at 37°C under shaking (120 rpm).

Moreover, the new isolate, resulting positive to the hydrolytic activity of PCL and tributyrin, was preliminary morphologically characterized by growth on the LB agar plate, and Gram stain examined under a microscope with a magnification up to 100X (Axiolab E re, Carl Zeiss, Germany). Oxidase and catalase tests were also performed according to standard protocols (Gaby and Hadley, 1957; Thomas, 1963).

### 2.3 Nucleic acid extraction and manipulation

The genomic DNA of bacterial isolates grown on LB was extracted following the manufacturer's instructions for the DNeasy UltraClean Microbial Kit (Qiagen, Italy).

The amplification of the 16S rRNA gene was performed using 2.5 U μl^−1^ of Long Range DNA Rabbit Polymerase (Eppendorf, Germany) with the following thermocycling conditions: at 95°C for 2 min, 95°C for 30 s, specific Tm (55°C) for 30 s, 72°C for 1 min and 45 s, for 30 cycles; and 72°C for 7 min. The universal bacterial primers 27 F and 1495 R utilized for the amplification are 27 F (AGAGTTTGATCCTGGCTCAG) and 1495 R (CTACGGCTACCTTGTTACGA) (Lane, 1991). An aliquot of the PCR product was verified on agarose gel (0.8%) using a 500–10,000 bp molecular ladder (Merck, Italy), and the purified PCR product was sequenced by Sanger sequencing. The full length of the 16S rRNA gene was obtained by comparing forward and reverse sequences, and the final gene fragment was then compared to the nucleotide BLAST (BLASTn) of NCBI (Altschul et al., 1990).

RNA extraction was performed using the RNA-Total RNA Mobio Isolation Kit (Qiagen Italia, Italy) according to the manufacturer's instructions. The extraction was performed from 100 ml or 50 ml of *R*. *erythropolis* D4 and *R. opacus* R7 cultures, respectively, grown on M9-PCL for 3 days or M9-M for 24 h at 30°C. The cells were harvested at the late-exponential growth phase (OD_600_ ~0.5 ± 0.05).

### 2.4 Growth of *Rhodococcus* strains on PCL

A biodegradative assay was performed to evaluate the ability of the *Rhodococcus* strains to grow and degrade PCL. Each *Rhodococcus* culture was established, as described in paragraph 2.1. At different times within a 42-day period (0, 1, 2, 3, 7, 14, 21, 28, and 42 days), each culture was sampled to evaluate the optical density and the total amount of bacterial cells using the CFU count method by serially diluting 0.1 ml of each bacterial culture in a solution of M9 medium and plating the diluted suspension on LB agar medium. The plates were incubated at 30°C for 48 h. Both OD_600_ and CFU ml^−1^ data are reported as the mean of three biological replicates with standard deviation (SD).

### 2.5 Biodegradative assessment of *Rhodococcus* strains on PCL by GC-MS analysis

PCL biodegradation by the two *Rhodococcus* strains was evaluated on the M9-PCL flask cultures as described in paragraph 2.1. Flasks added with M9 mineral medium and PCL and without inoculum were used as the controls. Biodegradation was recorded for up to 42 days, and the metabolic products of the properly extracted and derivatized samples were analyzed using gas-chromatographic analysis. Experiments were performed in biological triplicate. Biodegradation products were extracted from the *Rhodococcus* cultures with half a volume of DCM by manually shaking the separation funnel for 10 min and repeating the extraction twice. For the derivatization, aliquots of the extracted organic phase were mixed with N,O-bis(trimethylsilyl)-trifluoroacetamide (BSTFA) in a volume ratio BSTFA:sample of 1:3 and incubated at 60°C for 20 min. Derivatized extract samples (1 μl) were analyzed with a 6890 N Network gas chromatograph (GC) system (J&W DB-5ms 60 m × 0.25 mm, 0.25 μm Ultra Inert GC Column, Agilent Technologies, Santa Clara, CA, USA) with He at 99.99% as a carrier gas, coupled to a 5973 Network Mass Selective Detector (MSD, Agilent Technologies) at 70 eV in the scan ion monitoring mode (40–600 Da). The GC inlet temperature was 200°C. The analyses were carried out in split injection mode (split ratio 10.5:1) with the following temperature program: 3 min at 60°C, 10°C min^−1^ to 300°C, and holding this temperature for 6 min. The resulting chromatograms were interpreted using MSD ChemStation E.02.02.1431 (Agilent Technologies, Santa Clara, CA, USA). Unknown products were identified by comparing their mass spectra with the NIST11 database if their similarity to the reference mass spectra was greater than 90%.

### 2.6 Enzyme activity assay

The specific enzymatic activity of the cell-free culture supernatant (CFS) of *Rhodococcus* strains upon growth on M9-CPL or M9-M was determined spectrophotometrically using *p*NP-esters with different acyl chain lengths as substrate (Winkler and Stuckmann, 1979; Gupta et al., 2002). Each *Rhodococcus* culture was centrifuged at 7,000 g, and the supernatant was filtered with 0.45 μm filters (Millipore, Italy) to remove the residual PCL; subsequently, the CFS was lyophilized and resuspended in 1/50 of the initial volume of 10 mM potassium phosphate buffer pH 7.0.

The hydrolytic activity of concentrated CFS was tested against the following *p*NP-ester substrates: *p*-nitrophenyl acetate (*p*NPA, C2), *p*-nitrophenyl butyrate (*p*NPB, C4), *p*-nitrophenyl octanoate (*p*NPO, C8), *p*-nitrophenyl laurate (*p*NPL, C12), and *p*-nitrophenyl palmitate (*p*NPP, C16; Merck, Italy).

In 1 ml final volume, 50 μl of 20 mM substrates dissolved in isopropanol were mixed in 10 mM Tris-HCl pH 8, 1% TritonX-100. Hydrolysis of *p*NP-esters was carried out at 25°C and followed in continuous mode for 3 min by measuring the increase in absorbance at 405 nm with a JASCO V-770 UV/NIR spectrophotometer (JASCO Europe, Lecco, Italy). One unit (U) of enzyme activity is defined as the amount of enzyme catalyzing the formation of 1 μmol of *p*-nitrophenol per minute under the reported conditions.

The specific activity was calculated as U mg^−1^, and data are reported as the mean of three biological replicates with SD. The significant differences were assessed by Student's *t*-test, showing ^*^p-value < 0.05 or ^**^p-value < 0.01.

The total protein concentration of CFS was assessed using the Bradford method (Bradford, 1976) using Coomassie brilliant blue with bovine serum albumin as a standard.

### 2.7 Genome sequencing and preliminary analyses

Genome sequencing of *R*. *erythropolis* D4 was performed using Illumina MiSeq v3 (2 × 300 pb; Illumina Inc., San Diego, CA). The sequencing library was prepared using Nextera XT DNA Library Preparation Kit with the standard Illumina DNA shotgun library preparation protocol (Illumina Inc., San Diego, CA). *R. erythropolis* D4 genome sequencing resulted in 1.075 million reads.

The quality of sequencing was evaluated using FastQC v0.11.7 (Andrews, 2010) before and after the trimming process that was obtained using Trimmomatic v.0.38 (Bolger et al., 2014) to discard the sequences with per base sequence quality score <30. The retrieved sequences were then assembled into contigs using Spades 3.15.5 (Bankevich et al., 2012). All contigs were assembled into scaffolds using MeDuSa v1.6 (Bosi et al., 2015), and the complete genomes of the following *R*. *erythropolis* strains as reference: PR4 (NCBI BioProject PRJDA20395), CCM2595 (PRJNA81583), X5 (PRJNA573614), R85 (PRJNA703051), KB1 (PRJNA611840), R138 (PRJNA188397), D310-1 (PRJNA491403), CERE8 (PRJNA666739), BG43 (PRJNA280916), and JCM 2895 (PRJDB7038).

Genome assembly was evaluated by quality (completeness) and contiguity metrics at both contig and scaffold levels using QUAST 5.0.1 (Gurevich et al., 2013); this assessment was performed considering the total assembly length (expected to be ~6–7 Mb) the number and size of contigs/scaffolds, N50 (expected as large as possible), and NG50 (as large as possible).

The genome's preliminary annotation was performed using the Rapid Annotation using Subsystem Technology (RAST) server (Aziz et al., 2008). Gene prediction and annotation of D4 genome sequences were also automatically achieved using Prokka (v. 1.14.6) (Seemann, 2014). Protein coding genes were scanned for their organization into operons using the web server Operon-mapper (Taboada et al., 2018). The Comprehensive Antibiotic Resistance Database (CARD) and Resistance Gene Identifier (RGI; v. 5.2.1) were utilized to predict genes for antibiotic resistance (Alcock et al., 2020). CRISPR/Cas Finder (v. 4.2.20) was used for Clustered Regularly Interspaced Short Palindromic Repeats (CRISPR) search (Couvin et al., 2018), and Phigaro (v. 2.3.0) contributed to detect and annotate prophage regions (Starikova et al., 2020).

The whole-genome sequencing project has been deposited in the European Nucleotide Archive (ENA) under accession number PRJEB63643.

### 2.8 Whole-genome comparisons of *R. erythropolys* D4 and *R. opacus* R7

Sequence and functional comparisons were performed utilizing the RAST server between *R*. *erythropolis* D4 vs. *R*. *opacus* R7.

A BLAST comparison and the measure of nucleotide-level genomic similarity, average nucleotide identity (ANI) (Jain et al., 2018) were also calculated between the two *Rhodococcus* genomes using BLAST+ (v. 2.12.0) (Altschul et al., 1990) and FastANI (v. 1.3.3), respectively. Enzyme functions and metabolic pathways were predicted by the Kyoto Encyclopedia of Genes and Genomes (KEGG) database (Kanehisa et al., 2023) using the RAST server.

### 2.9 Bioinformatic clustering of protein sequences

With the aim of predicting the gene products potentially involved in PCL biodegradation of *R. erythropolis* D4 and *R. opacus* R7, we selected a set of reference amino acid (aa) sequences (RAS) derived from different microorganisms involved in PCL or polyester metabolism that were retrieved through a manual search of the literature and various databases (Table 1). These were 4ZV7 (Strzelczyk et al., 2015; Shi et al., 2020), BAI99230 (Hu et al., 2010), WP_004373894, WP_003239806 (Li et al., 2022), ADK73612 (Inglis et al., 2011), BAC67242 (Masaki et al., 2005), A0A0K8P6T7 (Yoshida et al., 2016), ADH43200 (Ribitsch et al., 2011), and OL660765 (Won et al., 2022). Two different approaches were pursued to identify the putative PCL catabolic enzymes (PCEs), both based on the similarity with RAS: (i) gene products annotated as lipases, esterases, hydrolases, or EC 3.1. were searched within the RAST server, and then they were compared and clustered by sequence alignment with RAS; (ii) the alignment of RAS was carried out against the two *Rhodococcus* genomes to directly identify gene products with the highest percentage of similarity.

**Table 1:** List of reference amino acid sequences (RAS) from different microorganisms involved in PCL or polyester metabolism deriving from a manual search of the literature and various databases.

**Name used for the clusterization**	**NCBI accession ID**	**UniProt accession ID**	**Gene accession number^a^**	**Protein accession number^a^**	**Type**	**Source**	**References**
R1	4ZV7^b^	P41365	Z30645	CAA83122	Chain A, lipase B	*Moesziomyces antarcticus*	Strzelczyk et al., 2015; Shi et al., 2020
R2	BAI99230	D4Q9N1	AB445476	BAI99230	Esterase	*Thermobifida alba*	Hu et al., 2010
R3	WP_004373894	A0A8I0SEY2	JADMNJ010000005	MBF8162059	Hypothetical protein	*Pseudomonas mendocina*	Li et al., 2022
R4	WP_003239806	A0A8I0SFQ4	JADMNJ010000002	MBF8160636	Triacylglycerol lipase	*Pseudomonas mendocina*	Li et al., 2022
R5	ADK73612	E9KJL1	GU592443	ADK73612	Cutinase A precursor	*Pseudomonas oleovorans*	Inglis et al., 2011
R6	BAC67242	Q874E9	AB102945	BAC67242	Cutinase-like protein	*Cryptococcus* sp. S-2	Masaki et al., 2005
R7	A0A0K8P6T7	A0A0K8P6T7	BBYR01000074	GAP38373	Poly(ethylene terephthalate) hydrolase	*Ideonella sakaiensis*	Yoshida et al., 2016
R8	ADH43200	D7R6G8	HM040886	ADH43200	Para-nitrobenzylesterase	*Bacillus subtilis*	Ribitsch et al., 2011
R9	OL660765	–	–	–	Cutinase	*Antarctic Rhodococcus* sp.	Won et al., 2022

^a^NCBI accession number, the ID derived from EMBL, GenBank, or DDBJ databases.

^b^4ZV7, the accession number derived from the PDB database.

All pairwise or multiple alignments have been carried out by the Clustal Omega program using the default parameters of the multiple sequence alignment (MSA) tool (Neighbor-Joining method, Gonnet transition matrix, 6-bit gap opening penalty, maintain gaps with 1-bit extension, bed-like clustering used during subsequent iterations, and zero number of combined iterations) (Sievers et al., 2011).

MSA was used for the inferred cluster analysis using the maximum likelihood (ML) method by MEGA (version 10.2) software (Kumar et al., 2018), with the following settings: JTT substitution matrix and gamma distribution of mutation rates with gamma optimized to 2. As an inferred phylogeny test, 50 bootstrap replicates were used.

The first approach generates a preliminary clusterization tree for each strain with clusters defined on the basis of the nine RAS. *Rhodococcus* gene products belonging to the clusters, including the references, were retrieved for subsequent evaluations.

The selected sequences deriving from the two separate strategies were compared by multiple alignments and a subtree clusterization for each *Rhodococcus* strain comprising the nine RAS in order to identify the potential target sequences.

The retrieved aa sequences of PCEs by the two approaches were then analyzed using SignalP (v. 6.0) for “other” organisms in the “fast” model (Teufel et al., 2022) to predict the presence and the location of aa translocation signal conferring the potential ability of these proteins to cross the cellular membrane. The following signals were predicted: Sec/SPI, secretory signal peptides transported by the Sec translocon and cleaved by Signal Peptidase I (Lep), Sec/SPII, lipoprotein signal peptides transported by the Sec translocon and cleaved by Signal Peptidase II (Lsp), Tat/SPI, Tat signal peptides transported by the Tat translocon and cleaved by Signal Peptidase I (Lep), Tat/SPII, Tat lipoprotein signal peptides transported by the Tat translocon and cleaved by Signal Peptidase II (Lsp), Sec/SPIII, and Pilin and pilin-like signal peptides transported by the Sec translocon and cleaved by Signal Peptidase III (PilD/PibD). Only the PECs possessing signal peptides were further considered for expression analyses.

### 2.10 Quantitative real-time PCR

The transcriptional levels of *R*. *erythropolis* D4 and *R*. *opacus* R7 genes potentially involved in PCL degradation were evaluated by RT-qPCR assays. The amplified genes encode hypothetical proteins, lipases, and 16S rRNA used as a reference.

Total RNA was reverse transcribed using an iScriptcDNA Synthesis kit (BIO-RAD, Italy), obtaining 200 ng of cDNA under the following thermocycling conditions: 5 min at 25°C followed by 1 h at 42°C and then 5 min at 85°C.

The cDNA thus obtained was quantitatively amplified by mixing 4.4 μl sample with 5 μl of PowerUp SYBR Green Master Mix (Applied Biosystem, Thermo Scientific, Italy) and 300 nM of each primer couple (Supplementary Table S1) in 10 μl final volume. The reactions were incubated in a StepOnePlus Real-Time PCR System (Applied Biosystem, Italy) using the following temperature program: 30 s at 95°C, followed by 40 cycles of 5 s at 95°C, 10 s at 60°C, and 45 s at 72°C and a melting curve cycle of 15 s at 95°C, 1 min at 60°C, and 15 s at 60°C. Expression levels of 16S rRNA were used as a reference (Bustin et al., 2009) to normalize gene expression according to the ΔΔCt method (Su et al., 2016). The growth condition in M9-M was used as a reference to determine the relative abundance of target transcripts. Moreover, DNA contamination was excluded by verifying that no amplification signal was obtained in parallel processed negative control samples to which reverse transcriptase was omitted under the same temperature program and primer sets for 35-cycle amplification. Data are expressed as the mean of three replicates ± SD.

## 3 Results

### 3.1 Isolation of bacterial strain from plastic and polyester-contaminated organic waste

A plastic-rich sludgy waste was sampled in the final step of an organic waste-treatment plant. The microbial characterization showed that the total viable count of heterotrophic bacteria grown on a solid LB medium was 2 × 10^5^ CFU ml^−1^. Enrichment cultures were established by 7-day growth in M9 medium added with plastic-rich sludgy waste. Around fifty individual bacterial colonies isolated on LB agar medium from enrichment cultures were subsequently pre-screened for their ability to hydrolyze tributyrin (C4), a non-specific substrate for esterases, and the potential ability to also hydrolyze PCL, a standard aliphatic biodegradable polyester (Molitor et al., 2020). Nine isolated bacterial strains produced a clear halo around their colonies on tributyrin (data not shown), and among them, only one isolate, namely D4, also produced a halo on plates containing PCL.

D4 strain colonies grown on LB agar plates were rough, opaque, light-yellow in color, and had round morphology (Supplementary Figure S1A). The characterization of the D4 strain showed that it is a Gram-positive, aerobic, catalase-positive, and oxidase-negative bacterium with short rod-shaped cells typical of coryneform bacteria (Supplementary Figure S1B). Morphology analyses and sequencing of 16S rRNA (Supplementary Figure S1C) of the D4 strain revealed that the new isolate belonged to *R. erythropolis* species with 99.6% similarity to *R. erythropolis*.

**Figure 1:**
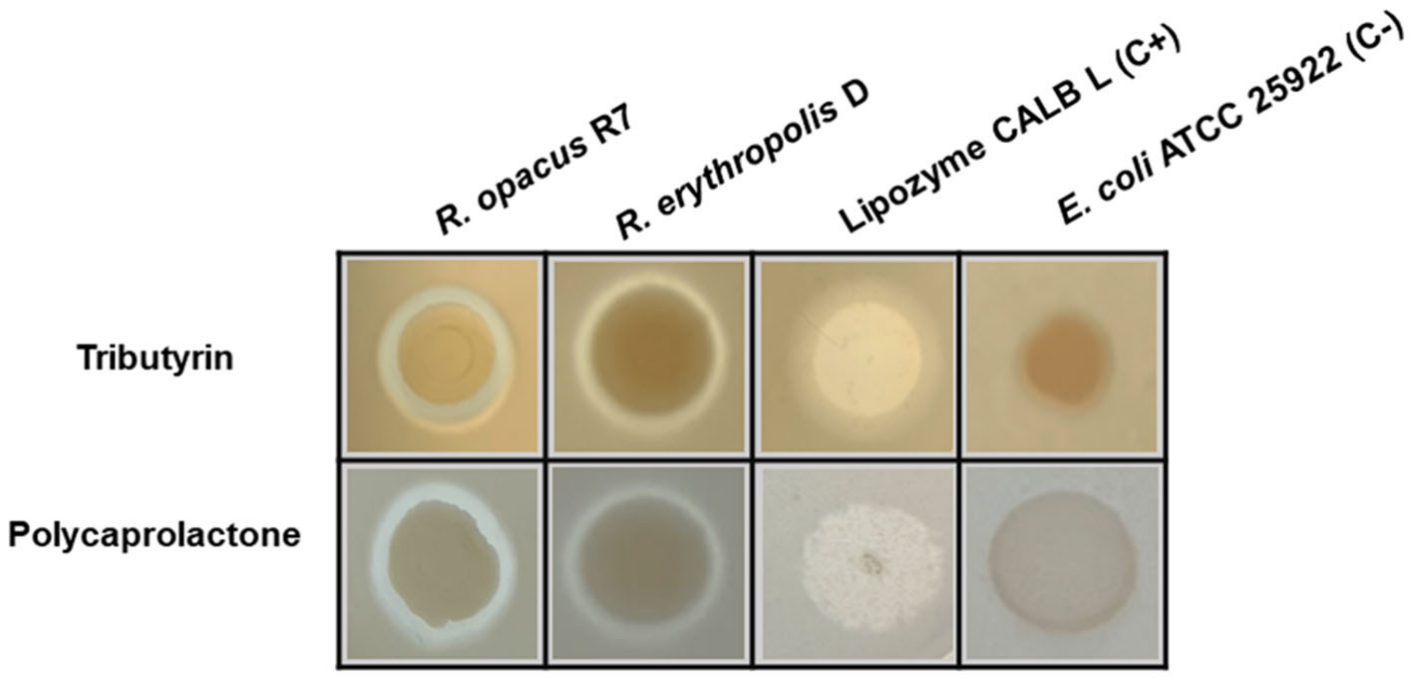
Halo assay on tributyrin and PCL agar plates for *Rhodococcus erythropolis* D4 and *Rhodococcus opacus* R7. Clarification halos around the cell spots indicate the hydrolytic activity. *Escherichia coli* ATCC 25922 cells were used as the negative control, and a lipase solution of Lipozyme CALB L served as the positive control.

Figure 1 shows the results of the preliminary screening for the hydrolytic activity of tributyrin and PCL. The hydrolytic activity of *R*. *erythropolis* D4 was compared with that of *R*. *opacus* R7, a strain with an extraordinary metabolic potential toward aliphatic and polycyclic aromatic hydrocarbons and plastic polymers (Orro et al., 2015; Zampolli et al., 2021), whose genome has already been sequenced (Di Gennaro et al., 2014). In this halo assay, *E*. *coli* ATCC 25922 cells served as the negative control, while a Lipozyme CALB L commercial preparation was used as the positive control (Oh et al., 2022; Rosato et al., 2022) (Figure 1). The evidence of hydrolytic activity on tributyrin and PCL prompted finer analyses of the ability of both *Rhodococcus* strains to metabolize PCL and use it as the only carbon and energy source.

### 3.2 *Rhodococcus* strain growth on PCL

**Figure 2:**
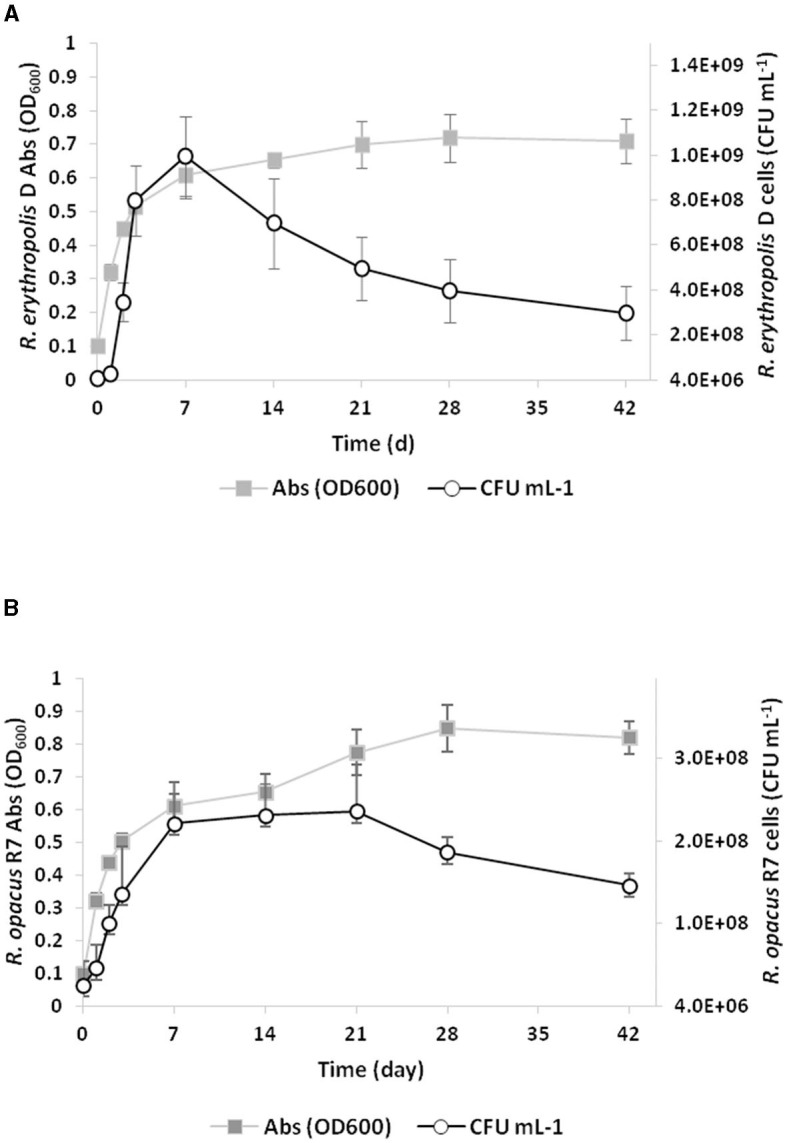
Growth curves of *Rhodococcus erythropolis* D4 **(A)** and *Rhodococcus opacus* R7 **(B)** on M9-PCL medium containing 1% PCL as the only carbon and energy source. Growth is reported as the mean value of the absorbance recorded at OD_600_ or counts of bacterial live cells expressed as CFU ml^−1^.

Based on the preliminary screening of tributyrin and PCL, PCL growth experiments were conducted by inoculating *Rhodococcus* strains in liquid M9-PCL medium and monitoring cell growth up to 42 days by both optical density at OD_600_ and cell viable count. Figure 2 shows the kinetic growth profile of *Rhodococcus* strains, evidencing that both strains have the capability to grow on PCL. *Rhodococcus erythropolis* D4 cell numbers doubled in 7 days, reaching the maximum live cell density of approximately 10^9^ CFU ml^−1^ (OD_600_ ~0.61; Figure 2A). After the first week, a drastic drop in viable count was observed while the OD_600_ density continued to increase. Therefore, 28 days after inoculation, the maximum OD_600_ (0.72 ± 0.07) corresponded to a significantly lower viable count (4.0 ± 1.4 × 10^8^ CFU ml^−1^) than what was observed within the first week. Over the same time period (7 days), *R*. *opacus* R7 initially showed an increase in cell numbers of a magnitude order (~ 2 × 10^8^ CFU ml^−1^) and OD_600_ value equal to what was observed for the D4 strain (Figure 2B). However, in the case of the R7 strain, the density of the live cells remained constant for up to 28 days, and then a slight decrease was registered. Since PCL supports the growth of *Rhodococcus* strains as the sole carbon and energy source, direct evidence of its catabolic degradation was sought.

### 3.3 Biodegradation of PCL by GC-MS analyses

PCL biodegradation was determined for each *Rhodococcus* strain by analyzing the residual PCL and its metabolic products extracted with organic solvent (DCM) from the exhausted cultures. The extracted compounds were then analyzed by GC-MS and compared with the chromatographic profile obtained on PCL powder not exposed to any (a)biotic degradation. Unprocessed, commercial PCL powder was found to contain cyclopentane carboxylic acid, decyl ester, hexadecanoic acid, octadecanoic acid, and glutaric acid ester derivative (Supplementary Figure S2A). After a 3-day incubation of each *Rhodococcus* strain in M9-PCL, only two new peaks were recorded, corresponding to pentanedioic acid (glutaric acid) and heptanoic acid (Table 2). The main differences due to the growth of *Rhodococcus* strains on PCL appeared 28 days after inoculation (Supplementary Figures S2B–D). With a few slight distinctions between *R*. *erythropolis* D4 and *R*. *opacus* R7, the main metabolic products were carboxylic acids with carbon chains between C9 and C20, hexanedioic acid, ketones (2-nonadecanone and 2-heptacosanone), and C19–C28 aliphatic hydrocarbons (Table 2; Supplementary Figures S2B–D). Interestingly, with time (42 days), the chromatographic profiles appeared similar to the initial times, with the exception of the R7 profile, which showed some carboxylic acids and alkyl products (Table 2; (Supplementary Figures S2E–G). Overall, PCL appeared to be degraded by both *Rhodococcus* strains through the action of hydrolytic enzymes.

**Table 2:** Compounds detected by GC-MS analyses deriving from PCL powder biodegradation by *Rhodococcus erythropolis* D4 and *Rhodococcus opacus* R7.

**Compound^a^**	**Retention time (*t*_R_) (min)**	**Molecular Weight**	**Formula**	**CAS number**	**t0**	***Rhodococcus erythropolis*** **D4**			***Rhodococcus opacus*** **R7**			**No inoculum**		
						**3 days** ^b^	**28 days**	**42 days**	**3 days**	**28 days**	**42 days**	**3 days**	**28 days**	**42 days**
Nonanoic acid	15.03	158	112-05-0	C_9_H_18_O_2_	–	–	+	–	–	±	–	–	–	–
Pentanedioic acid (glutaric acid)	15.85	132	110-94-1	C_5_H_8_O_4_	–	+	–	+	+	+	+	+	+	+
Decanoic acid	16.33	172	334-48-5	C_10_H_20_O_2_	–	–	+	–	–	+	–	–	–	–
Hexanedioic acid	16.90	146	124-04-9	C_6_H_10_O_4_	–	–	+	–	–	+	+	–	–	–
Undecanoic acid	17.55	186	112-37-8	C_11_H_22_O_2_	–	–	+	–	–	–	–	–	–	–
Dodecanoic acid	18.74	200	143-07-7	C_12_H_24_O_2_	–	–	+	–	–	+	±	–	–	–
*n*-Tridecanoic acid	19.85	214	638-53-9	C_13_H_2_6O_2_	–	–	+	–	–	+	–	–	–	–
Cyclopentanecarboxylic acid, decyl ester	20.22	254	100028-04-1	C_16_H_32_O_2_	+	+	–	+	+	+	+	+	+	+
1,4-Benzenedicarboxylic acid	20.33	166	100-21-0	C_8_H_6_O_4_	–	–	+	–	–	+	+	–	+	–
Tetradecanoic acid	20.91	228	544-63-8	C_14_H_28_O_2_	–	–	+	–	–	+	+	–	–	–
*n*-Pentanoic acid	21.92	102	109-52-4	C_5_H_10_O_2_	–	–	+	–	–	+	–	–	–	–
Hexadecanoic acid	22.90	256	57-10-3	C_16_H_32_O_2_	+	+	+	+	+	+	+	+	+	+
Heptanoic acid	23.97	130	111-14-8	C_7_H_14_O_2_	–	+	–	–	+	–	–	–	–	–
2-Hexadecanone	24.47	240	18787-63-8	C_16_H_32_O	–	–	–	–	–	+	–	–	–	–
Octadecanoic acid	24.72	284	57-11-4	C_18_H_36_O_2_	+	+	+	+	+	+	+	+	+	+
Eicosane	25.25	283	112-95-8	C_20_H_42_	–	–	+	–	–	+	+	–	–	–
Nonadecanoic acid	25.57	299	646-30-0	C_19_H_38_O_2_	–	–	–	–	–	+	–	–	–	–
Tetracosane	26.10	339	646-31-1	C_24_H_50_	–	–	+	–	–	+	–	–	+	+
2-Docosanone	26.20	184	77327-11-8	C_12_H_24_O	–	–	–	–	–	+	–	–	–	–
Eicosanoic acid	26.39	313	506-30-9	C_20_H_40_O_2_	–	–	–	–	–	+	–	–	–	–
Pentacosane	26.90	353	629-99-2	C_25_H_52_	–	–	+	–	–	+	+	–	–	+
Glutaric acid ester derivative^c^					+	+	–	+	+	–	+	+	+	+
Hexacosane	27.70	367	630-01-3	C_26_H_54_	–	–	+	–	–	–	+	–	–	+
Heptacosane	28.60	381	593-49-7	C_27_H_56_	–	–	+	–	–	+	+	–	–	–
Octacosane	29.59	395	630-02-4	C_28_H_58_	–	–	+	–	–	–	+	–	–	–
2-Heptacosanone	32.38	395	7796-19-2	C_27_H_54_O	–	–	–	–	–	+	–	–	–	–

^a^Compound, the considered degradation products had more than 90% similarity compared with the mass spectra of NIST11.

^b^3 days, number of days of incubation.

^c^+, the symbol “+” indicates the products, while “–” indicates the absence.

^d^Glutaric acid ester derivative—the mass spectra were similar only at 50% compared to the NIST database.

We then tested whether exposure to PCL caused overexpression of secreted esterases/lipases in the culture medium.

### 3.4 Hydrolytic activity assays on *Rhodococcus* strains

A direct spectrophotometric enzymatic assay was used to evaluate the ability of the *Rhodococcus* strains to secrete hydrolytic enzymes for the hydrolysis of aliphatic polyesters. The assay used *p*NP-esters with carbon chain lengths from 2 to 16 C as substrates, and a malate reference condition was used for comparison.

After 3 days of growth on M9-PCL medium, *Rhodococcus* cultures CFS were clarified and freeze-dried before being resuspended in 1:50 of the initial volume of 10 mM potassium phosphate buffer, pH 7.

**Figure 3:**
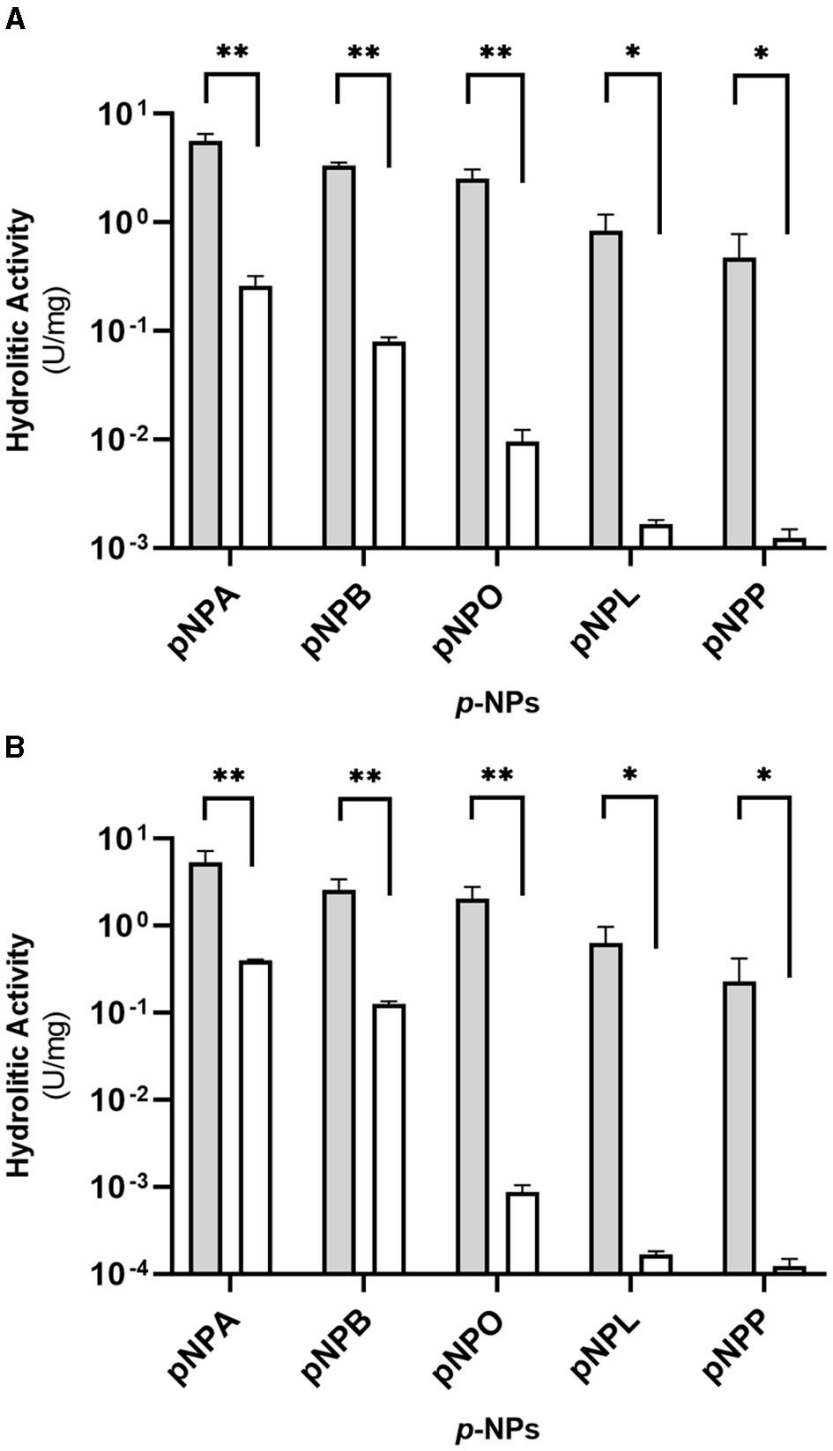
Hydrolytic activity (U mg^−1^ of total proteins) of the supernatants (CFS) of *Rhodococcus erythropolis* D4 **(A)** and *Rhodococcus opacus* R7 **(B)** against *p*NP esters with different acyl chain lengths after growth on M9-PCL (gray) or M9-M (white) used as the control. The hydrolytic activity is the mean of three replicates ± standard deviation. Statistical differences were calculated using the Student's *t-*test: *p-value < 0.05, **p-value < 0.01.

Figures 3A, B show the esterase activity secreted by *R. erythropolis* D4 and *R. opacus* R7 expressed in terms of specific activity. The highest activity was recorded in the presence of *p*NPA (~5.5 U mg^−1^), and it decreased proportionally with increasing acyl chain length of *p*NP substrates, showing the lowest activity on *p*NPP (< 0.5 U mg^−1^). Moreover, the results showed that the enzymatic activity was considerably high in the presence of *p*NPB and *p*NPO, besides *p*NPA. For both strains, the esterase activity of CFS from M9-PCL cultures in the presence of all tested *p*NP-esters was significantly different with respect to M9-M cultures (*p*-value < 0.05 in the presence of *p*NPL and *p*NPP substrates and *p*-value < 0.01 for *p*NPB, *p*NPO, and *p*NPA). The hydrolytic activities measured for the two strains of *Rhodococcus* spp. in the CFS are comparable. This suggests that the secreted carboxylesterase activities may mediate PCL catabolism.

This indication led to the search for genes coding for hydrolytic enzymes presumably involved in the catabolism of the PCL (PCEs) that was carried out through functional genomic analyses of the two *Rhodococcus* strains.

### 3.5 Genome sequencing of *R*. *erythropolis* D4 and comparison with *R. opacus* R7 genome

The genome of *R. erythropolis* D4 was completely sequenced by Illumina MiSeq v3 (2 × 300 bp), obtaining 1.075 million reads. The sequencing quality by FastQC showed that the shortest reads were ~280 bp long, and the average genome coverage was 99X. After read trimming using Trimmomatic (Bolger et al., 2014), the remaining reads with 40X coverage were assembled into 77 contigs using Spades 3.15.5 (Bankevich et al., 2012), with an *N*50 length of 284,698 bp. Contigs were assembled into 28 scaffolds by MeDuSa v1.6 (Bosi et al., 2015) using 10 reference genome sequences, resulting in a genome size of 6,381,126 bp and 62% G–C content.

Automated annotation analysis of *R*. *erythropolis* D4 genome sequences using RAST server (Aziz et al., 2008) identified a total of 6,165 open reading frames (ORFs) and 72 RNAs genes (6 rRNAs and 66 tRNAs), in agreement with the results obtained from the automated gene prediction and annotation software Prokka (Grant et al., 2023) that indicated 6,113 ORFs and 2,984 hypothetical proteins (HP)/putative proteins.

**Figure 4:**
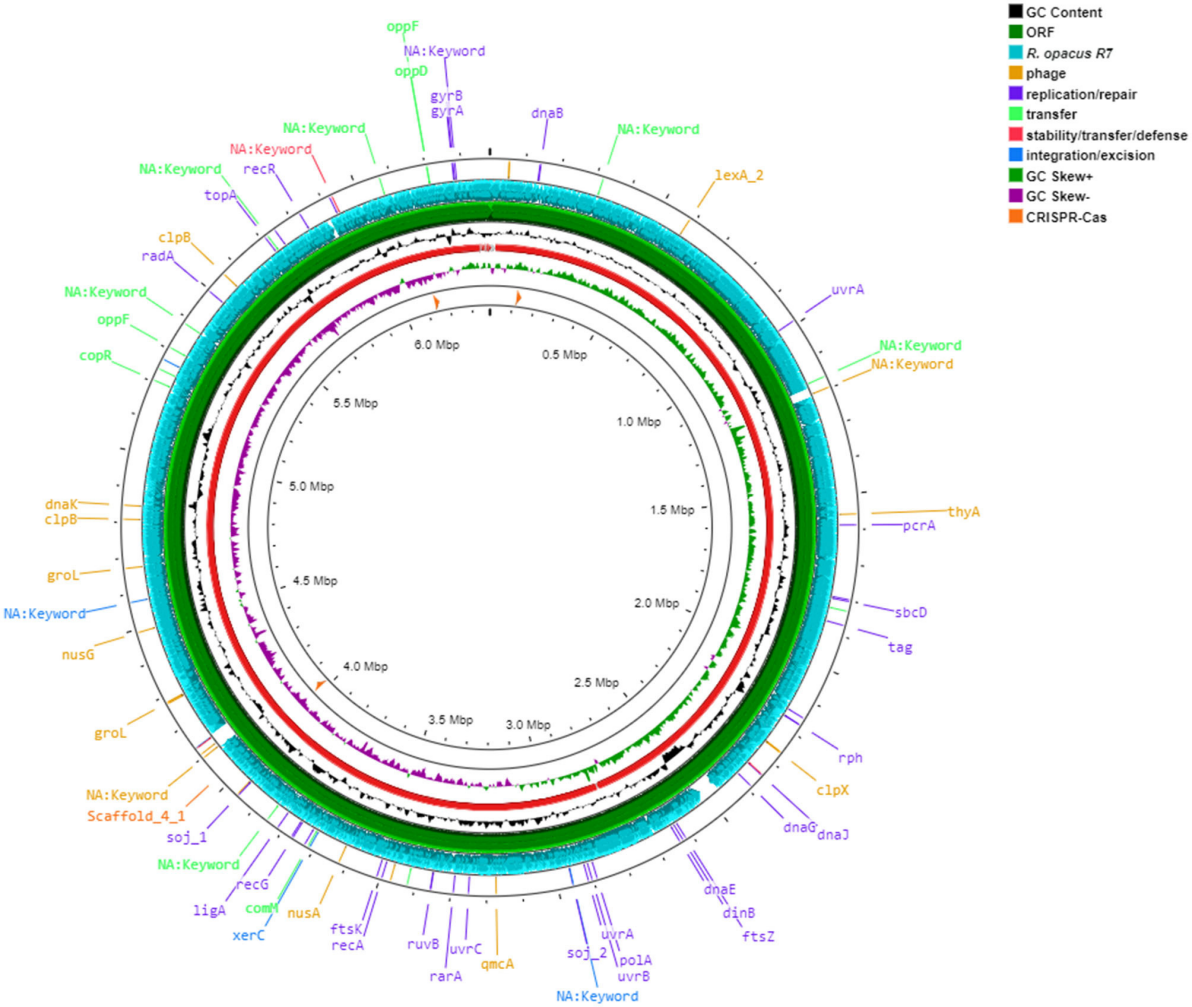
Circular map of *Rhodococcus erythropolis* D4 genome predicted with Proksee viewer (https://proksee.ca/) (accessed May 5, 2023). Successive rings from the outermost: ring 1, Mobile genetic elements annotation with Mobile OG DB marking *hsdR* gene involved in stability/transfer/defense, antibiotic resistance genes, and prophage regions; ring 2, comparison with CDS of *Rhodococcus opacus* R7 genome (light blue); ring 3, CDS of *R. erythropolis* D4 with Prokka annotation (green color); ring 4, GC content plot (black); ring 5, *R. erythropolis* D4 scaffolds (red); ring 6, GC skew information positive strand in green color and negative in purple color; and ring, 7 CRISPR-Cas sequences (orange color).

**Figure 5:**
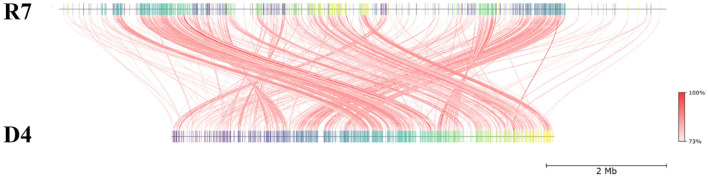
Pairwise comparison of *Rhodococcus erythropolis* D4 and *Rhodococcus opacus* R7 by whole-genome average nucleotide identity (ANI). R7: linear representation of *R. opacus* R7 genome; D4: linear representation of *R. erythropolis* D4 genome. The red lines indicate the correspondence between similar regions of the two genomes (95% threshold), thus representing the relatedness of the genome sequence. The heat map represents the ANI value for each orthologous match.

The draft genome of *R*. *erythropolis* D4 and its main features (GC content, ORFs, genes encoding for phages, replication or repair, transfer, stability/defense, integration and excision, and CRISPR-Cas) are represented in Figure 4. For the sake of comparison, the *R. opacus* R7 genome (light blue) was visualized with the same Proksee system. The ANI (Jain et al., 2018), indicating the nucleotide-level genomic similarity between *R. erythropolis* D4 and *R. opacus* R7 genomes, was equal to 79 with 800 orthologous matches (Figure 5). The comparative analyses were also inferred using the RAST server, showing that the closest neighbors of the *R. erythropolis* D4 genome were *Rhodococcus jostii* RHA1 (score 547) and *Nocardia farcinica* IFM 10152 (score 524).

Sequence-based and functional comparisons were also carried out between the two rhodococci, showing that 400 genes shared more than 90% identity with RAST, and 100 sequences shared more than 90% identity with BLAST. According to RAST analysis, 50% of identity was shared by 3,809 genes, accounting for 62% of ORFs of the D4 genome. These shared genes probably constituted a common “genomic core” that was accompanied by 19% of ORFs from the D4 genome, which were unique with respect to the R7 genome.

**Figure 6:**
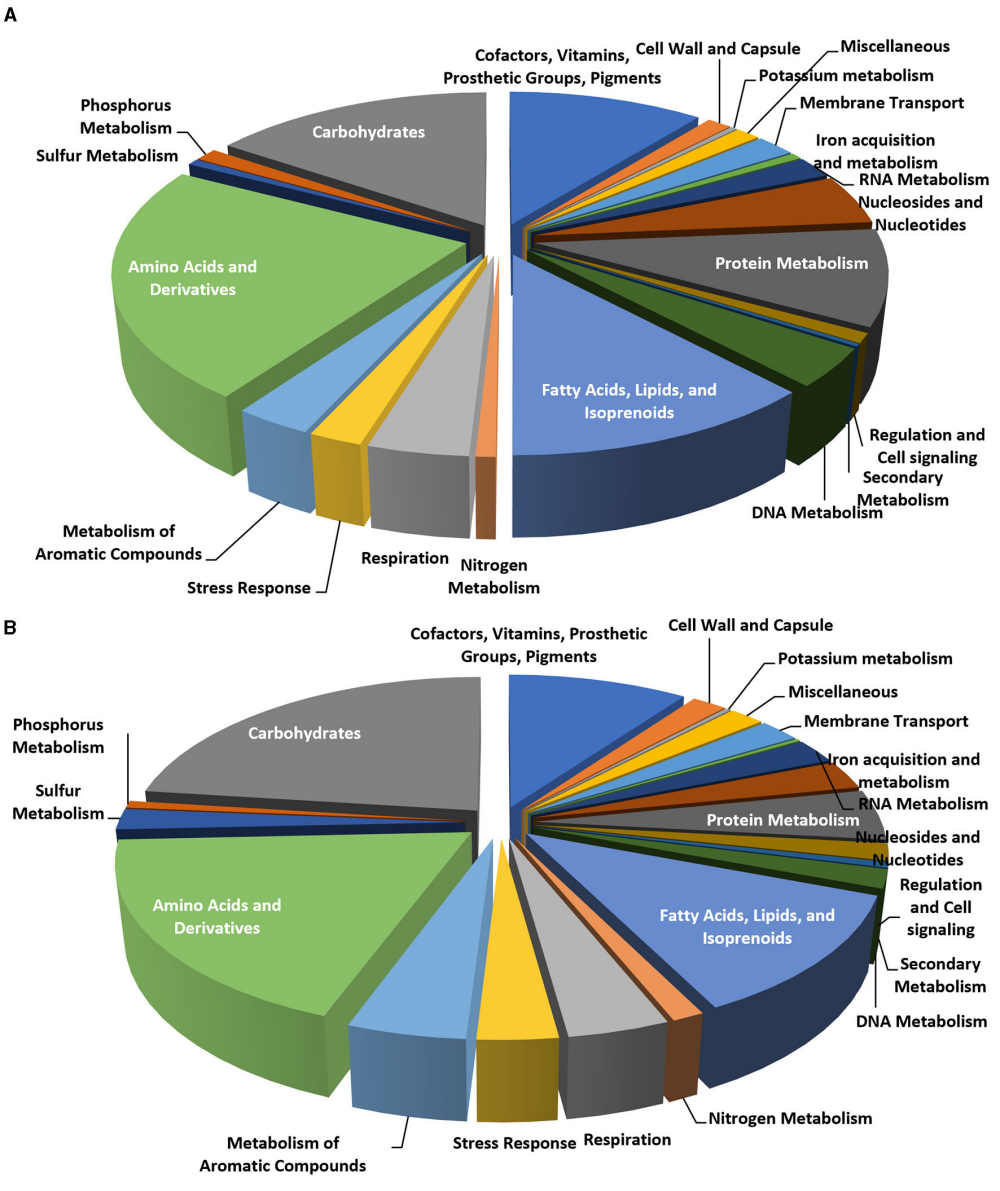
Pie charts representing the number of CDS in each of the RAST subsystem categories for *Rhodococcus erythropolis* D4 **(A)** and *R*. *opacus* R7 **(B)** genomes.

The analysis of the genome subsystems (Overbeek et al., 2005), carried out with the RAST server, indicated that out of 322 functional categories, the most populated were the same for the two *Rhodococcus* genomes, including “amino acids and derivatives,” “carbohydrates,” “fatty acids, lipids, and isoprenoids,” “cofactors, vitamins, prosthetic groups, or pigments,” and “protein metabolism” accounting for 510, 346, 272, 240, and 209 CDSs, respectively (Figures 6A, B).

Considering the possible role in aliphatic polyester degradation of genes involved in lipid metabolism, this functional category was analyzed in more detail. We considered various modules of lipid and carbohydrate metabolic pathways in the KEGG database. As a result, a diagram of the molecular interaction/reaction network involving clusters of orthologous genes was obtained so that the relationships experimentally described for a specific organism could be generalized to other organisms. Diverse EC numbers were associated with these genes for each functional module, including the following EC: 6.2.1.3, 1.3.3.6, 1.3.99.2, 4.2.1.17, 1.1.1.35, 2.3.1.16, 2.3.1.9, 2.3.1.16, 5.1.2.3, 1.1.1.1, 1.2.1.3, and 1.14.15.3. Table 3 summarizes the statistics of EC numbers from the KEGG metabolic maps based on automatically annotated genomes of *R. erythropolis* D4 and *R. opacus* R7. Both *Rhodococcus* strains showed a similar distribution of genes within the considered KEGG modules, while, as expected, most of the enzymes involved were found to be lipases and esterases. We focused our subsequent analyses on these types of gene functions.

**Table 3:** Categories of lipids and carbohydrate metabolism derived from the KEGG classification identified in the genome of *Rhodococcus erythropolis* D4 and *Rhodococcus opacus* R7.

**KEGG map**	**Distinct ECs^a^**	***Rhodococcus erythropolis* D4**	***Rhodococcus opacus* R7**
Citrate cycle (TCA cycle)	22	14^b^ (63.6%)^c^	16 (72.7%)
Fatty acid biosynthesis	21	12 (57.1%)	14 (66.7%)
Fatty acid elongation in mitochondria	8	3 (37.5%)	3 (37.5%)
Fatty acid metabolism	29	11 (37.9%)	16 (55.2%)
Glycerolipid metabolism	36	9 (25.0%)	10 (27.8%)
Synthesis and degradation of ketone bodies	6	4 (66.7%)	5 (83.3%)

^a^Distinct Ecs, the number of total EC for each selected KEGG metabolic map analyzed on the RAST server.

^b^Number outside the brackets, number of EC found in each genome for each selected KEGG metabolic map.

^c^Percentage in brackets, percentage of the number of EC found in each genome with respect to the total number for each selected KEGG metabolic map.

### 3.6 Genome mining for the discovery of PLC degradative genetic determinants of *Rhodococcus* strains by clusterization and whole-genome comparison with reference gene products

Genes potentially associated with PCL biodegradation present in *R. erythropolis* D4 and *R. opacus* R7 were bioinformatically predicted through two approaches. (i) Searching for gene products annotated by the RAST server specifically as lipase, esterase, hydrolase, or associated with EC number (3.1.-) and subsequently clustering them into phylogenetic trees using nine RAS (Table 1). (ii) Alignment of the nine RAS with the two *Rhodococcus* genomes and the identification of the gene products with the highest similarity percentage. All the sequence alignments were carried out by Clustal Omega.

The first approach allowed the retrieval of 29 and 33 lipases, 69 and 61 esterases, 116 and 190 hydrolases, and 112 and 155 gene products with EC number (3.1.-), respectively, from the genome of *R. erythropolis* D4 and *R. opacus* R7. Although the sequences in the latter category partially overlap those of the other three categories, each gene was considered only once in a non-redundant database in which the nine RAS were also included to generate a preliminary tree with the MEGA software (v. 10.2) software (Kumar et al., 2018).

Four and five clusters emerged in the tree of *R. erythropolis* D4 and *R*. *opacus* R7, respectively (data not shown). These clusters display 15 representative gene products of *R. erythropolis* D4 and 12 representatives of *R*. *opacus* R7, which were selected for further analysis.

By applying the second approach, i.e., the direct alignment of RAS with D4 and R7, respectively, 10 and 9 genes encoding hypothetical proteins and lipases were retrieved, with the highest sequence similarity ranging between 24 and 40%.

**Figure 7:**
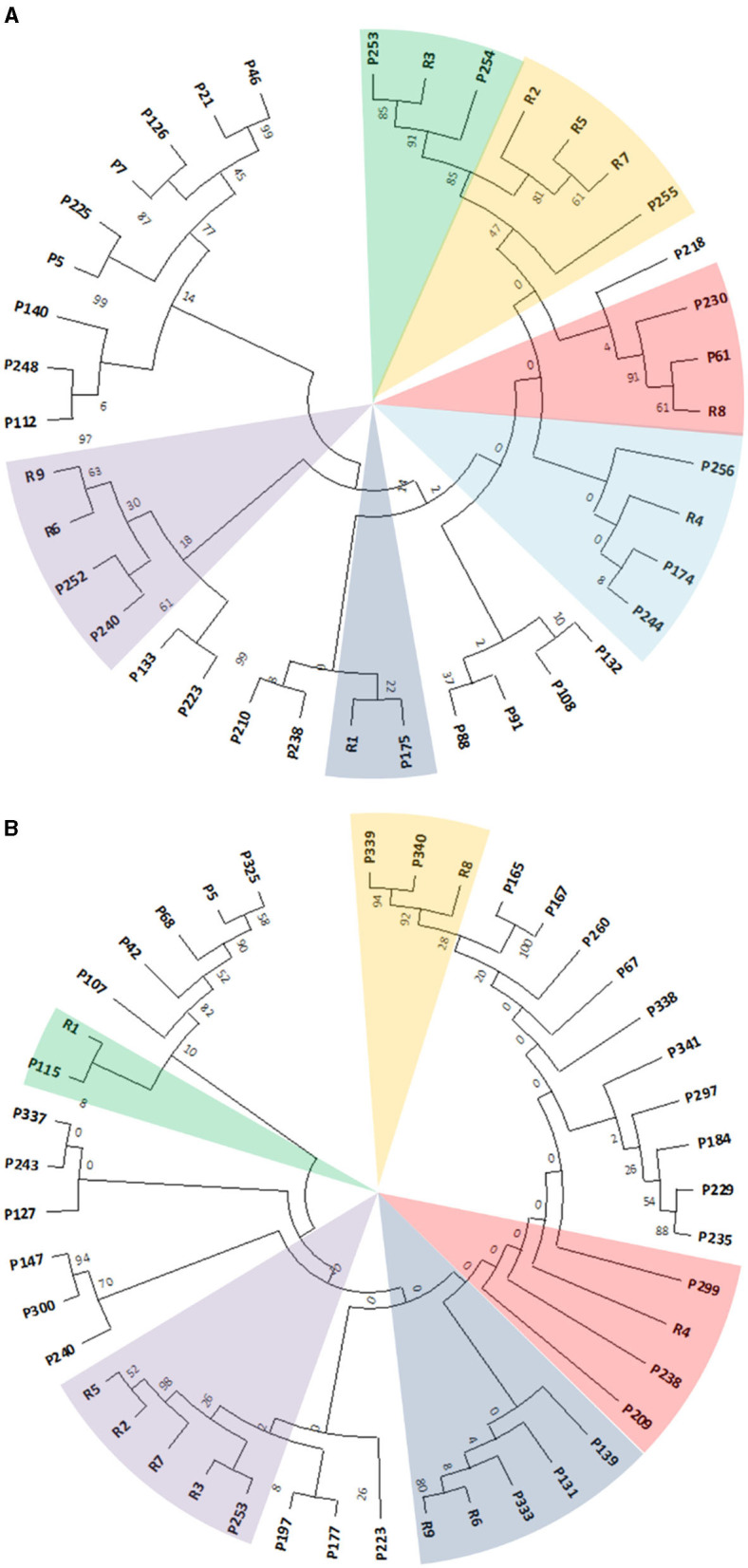
Subtree clusterization of selected gene products (named as P) from *Rhodococcus erythropolis* D4 **(A)** and *Rhodococcus opacus* R7 **(B)** genomes against nine RAS (named as R). The different sectors of the pie highlight the clusters identified through two genomic filtering approaches. The numbers on the branches represent the bootstrap values calculated for the maximum likelihood (ML) method from the MEGA software with 50 bootstraps. Protein name abbreviations are reported in Supplementary Tables S3, S4 for *R. erythropolis* D4 and *R. opacus* R7, respectively.

To obtain an overall overview of their clustering distribution and to manually select the gene products showing the highest similarity, all the identified putative genetic determinants were clustered in one tree for each *Rhodococcus* strain before focusing on the retrieved gene products from the two approaches. This was done to exclude the gene products that were excluded by using the single approaches but clustered with the RAS (Supplementary Figures S3, S4, Supplementary Tables S2, S3). Due to the expected complexity of the resulting clustering trees, only the selected sequences deriving from the two independent approaches and the ones retrieved by the overall multiple alignments were compared, and a single subtree for each *Rhodococcus* strain was generated (Figures 7A, B). *Rhodococcus erythropolis* D4 subtree constituted 29 selected sequences distributed in six main clusters comprising RAS, while *R. opacus* R7 subtree showed 34 selected sequences distributed in five main clusters.

These subtrees were used to identify the potential target sequences with the highest similarity to RAS, which was then analyzed for translocation signal prediction using SignalP software to ensure the successful crossing of the lipid bilayer by the folded proteins. Only the sequences possessing signal peptides were considered for further expression analyses and possible induction following PCL exposure. Specifically, all the considered sequences possessed Sec/SPI signals.

### 3.7 Expression analysis of selected genes by RT-qPCR after *Rhodococcus* strain exposure to PCL

**Figure 8:**
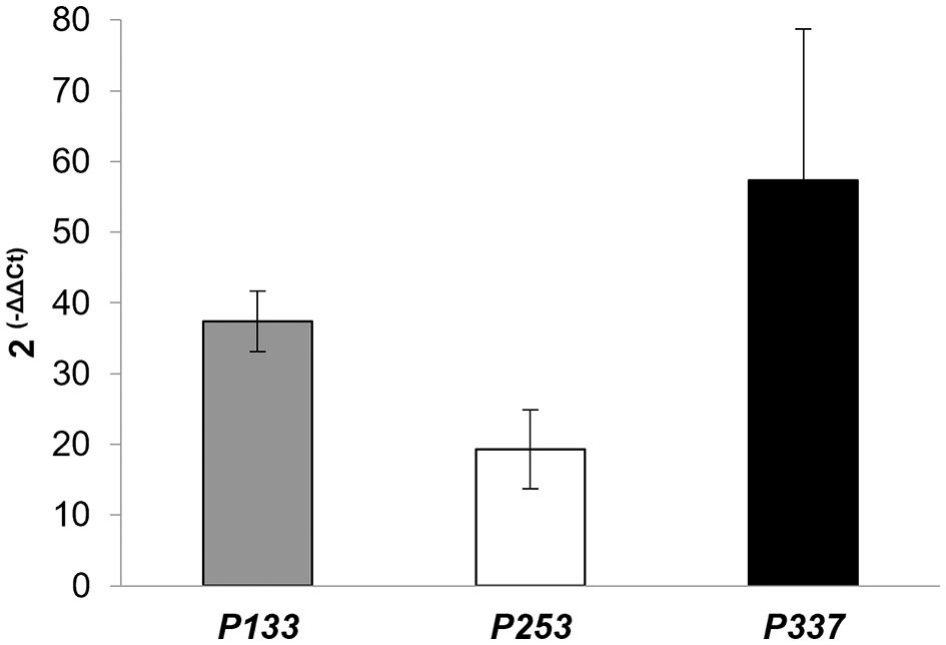
Expression levels of *P133* (encoding a hypothetical protein) and *P253* (encoding a lipase) of *Rhodococcus erythropolis* D4, and *P337* (encoding a hypothetical protein) of *Rhodococcus opacus* R7, assessed by RT-qPCR experiments. Data are expressed as mean values of three replicates ± standard deviation.

According to the genome-based analysis of *Rhodococcus* strains to predict potential genetic determinants for PCL degradation, gene expression was conducted by RT-qPCR to evaluate whether some of the selected putative gene products of *R. erythropolis* D4 and *R. opacus* R7 were expressed throughout the PCL biodegradation process. The selected genes encoded various types of lipases, such as probable triacylglycerol lipases, putative lipases, secreted lipases, and lipases with EC 3.1.1.3. Additionally, GTP cyclohydrolase I (EC 3.5.4.16) type 1 and genes that encoded hypothetical proteins were present for the D4 strain. As for the R7 strain, lipases LipV (EC:3.1.1.-), possible triacylglycerol lipases, probable lipases, and a hypothetical protein were detected. Samples of RNA were extracted from the 3-day cultures of each *Rhodococcus* strain grown in liquid M9-PCL medium or grown on M9-M as the basal expression level of PCL-inducible genes. After the cDNA synthesis, each gene was subjected to RT-qPCR and quantified according to the ΔΔCt method. Results are shown in Figure 8, indicating that the *P133* and *P253* genes, encoding respectively a hypothetical protein and a putative lipase in *R. erythropolis* D4, and the *P337* gene, encoding a hypothetical protein in *R. opacus* R7, showed the highest transcription values equal to 37 ± 4-fold, 19 ± 6-fold, and 57 ± 21-fold compared with the malate condition.

For completeness, these target gene products were pairwise aligned against the 9 RAS, showing that their aa identity varies between 18 and 37% (Table 4). The highest aa identity for P133 and P253 of *R. erythropolis* D4 was 26 and 37% against R6 (BAC67242) encoding a cutinase and R3 (WP_004373894) encoding a hypothetical protein (designated PCLase I), respectively. For P337 of *R. opacus* R7, the highest aa identity was 26% against R4 (WP_003239806), encoding a triacylglycerol lipase (designated PCLase II).

**Table 4:** Amino acid identity between the nine selected reference gene products and the gene targets involved in PCL degradation.

**Reference number**	**Reference ID^a^**	**Function**	***Rhodococcus erythropolis*** **D4**		***Rhodococcus opacus*** **R7**	
			**Number**	**aa identity (%)**	**Number**	**aa identity (%)**
R1	4ZV7^b^	Lipase	P133	22.9	P337	24.1
			P253	24.7		
R2	BAI99230	Esterase	P133	21	P337	22.2
			P253	26.1		
R3	WP_004373894	PCLase I	P133	24.3	P337	20.8
			P253	36.8		
R4	WP_003239806	PCLase II	P133	21.4	P337	25.8
			P253	20.8		
R5	ADK73612	Cutinase	P133	20.3	P337	20
			P253	25.4		
R6	BAC67242	Cutinase	P133	25.9	P337	22.3
			P253	26.2		
R7	A0A0K8P6T7	PTEase – Lipase	P133	19.2	P337	18.2
			P253	24.6		
R8	ADH43200	*para*-Nitrobenzylesterase	P133	21.1	P337	20.1
			P253	21.6		
R9	OL660765	Cutinase	P133	19.3	P337	23.1
			P253	23.9		

^a^NCBI Accession number, the ID derives from EMBL, GenBank, or DDBJ databases.

^b^4ZV7, the accession number derives from the PDB database.

## 4 Discussion

The issue of plastic waste has been a major concern for environmentalists and health experts alike. While the production of biodegradable polymers has offered a partial solution to this problem, it does not entirely solve the issue of waste management and the ensuing pollution. Regardless, the reduction or elimination of these biodegradable plastic polymers is desirable, and the exploitation of microbial degrading activity can offer an eco-friendly alternative to the conventional methods to accelerate degradation rates and avoid the accumulation of toxic compounds in the soil (Mo et al., 2023). Among others, PCL represents a model polyester to study the biodegradation process through the identification of degrading microorganisms and their involved enzymatic functions (Ma et al., 2020; Kosiorowsk et al., 2021). Microbial enzymes employed for the effective biodegradation of aliphatic polyesters possess physicochemical characteristics that influence the optimal degradative yields; thus, the study of their degradative capacities and new approaches are still ongoing (Gricajeva et al., 2021). However, the microbial contribution can guarantee a mineralization process with a low toxic by-product generation despite the competitive enzymatic processes in terms of time and industrial scale-up.

Members of the *Rhodococcus* genus are well-known for their degrading features against emerging contaminants (Alvarez, 2019), and they can be considered useful not only for biotechnological applications in the field of bioremediation but also for understanding the molecular and biochemical aspects of plastic degradation (Zampolli et al., 2022).

The present study aims to characterize PCL biodegradation by two *Rhodococcus* bacteria to correlate this capacity to their genomic traits and exploit their metabolic potential, acknowledging the functional aspects. In this study, two bacterial strains were taken into consideration—a new bacterial strain isolated by sampling plastic-rich organic waste and identified as *R*. *erythropolis* strain D4 and *R*. *opacus* R7, a prominent plastic-contaminant degrading *Rhodococcus* (Orro et al., 2015; Zampolli et al., 2020, 2021). *Rhodococcus* strains isolated from specific and sometimes harsh environments can develop unique features that help them thrive under unfavorable conditions (Urbanek et al., 2017; Auta et al., 2018). To the best of our knowledge, this is the first study that describes a genomic functional investigation on *Rhodococcus* biodegradative features on aliphatic polyesters correlating experimental degradative aspects and genome and genetic traits. Moreover, literature data report only two other PCL-degrading *Rhodococcus* whose functional features are not investigated in-depth (Urbanek et al., 2017; Won et al., 2022).

In general, the biodegradation of PCL by a microorganism is often demonstrated through a number of hallmarks that include the appearance of a clear halo around a cell spot on agar plates (Molitor et al., 2020) or other direct evidence of the production/secretion of enzymatic activity, mainly esterase, lipase, and cutinase (Gricajeva et al., 2021), the ability to metabolize PCL, i.e., to sustain the increase in biomass of microbial culture, as demonstrated by the increase in dry weight (Nawaz et al., 2014), and optical density (Chua et al., 2013).

Both *R. erythropolis* D4 and *R. opacus* R7 proved capable of degrading and metabolizing PCL, using it as their sole source of carbon and energy. Growth assays in M9 containing PCL indicate that both strains reach the maximum cell density in 1 week (1.1 ± 0.2 × 10^9^ CFU ml^−1^ and 2.2 ± 0.3 × 10^8^ CFU ml^−1^ for *R. erythropolis* D4 and *R. opacus* R7, respectively).

Interestingly, direct evidence of PCL depolymerization by the two *Rhodococcus* strains comes from GC-MS analysis of the culture medium and consists of the increase in carboxylic acids, which have been observed for the two strains as early as the first week of growth, but much greater after 1 month. It should be noted that the presence of carboxylic acid has a sinusoidal trend on both strains; this can be explained especially for *R*. *opacus* R7 since it is well-known for its capability to mineralize *n*-alkanes and carboxylic acids (Zampolli et al., 2014, 2020).

The appearance of carboxylic acids is compatible with the action of a hydrolytic enzyme, i.e., esterase, lipase, and cutinase, on aliphatic polyesters (Hakkarainen, 2003; Gricajeva et al., 2021). Consistently, these types of hydrolytic enzymes were found to be activated in the culture medium of both *Rhodococcus* spp. The hydrolysis of various *p*NP esters, with acyl chains from C2 to C16, could reflect the overexpression of multiple acyl ester hydrolases. Specifically, lipase overproduction is suggested by the detection of activity on longer-chain esters, i.e., acyl chain >C10 (Griebeler et al., 2011), and is corroborated by RT-qPCR data for at least one gene from the genome of the newly sequenced strain of *R*. *erythropolis* D4.

For this purpose, for the first time, a genome mining of *Rhodococcus* members was conducted to investigate the genetic determinants for PCL degradation to correlate with the phenotypic activities. Firstly, the genome of *R*. *erythropolis* D4 was sequenced, assembled, and analyzed. The size and percentage of GC of the genome were similar to that of the other strains of this species (https://www.ncbi.nlm.nih.gov/genome/browse/#!/prokaryotes/1638/). However, it was moderately different (Jain et al., 2018) from the genome of *R. opacus* R7 (Di Gennaro et al., 2014). Indeed, only 62% of ORFs from the D4 genome appeared to be the core repertoire shared with the R7 genome at 50% identity, according to functional comparison by the RAST server.

The potential genetic determinants for PCL metabolism were sought for the two diverse *Rhodococcus* species strains based on whole-genome alignment, automated annotation, similarity to reference hydrolytic enzymes, and prediction of their extracellular export. The combining of multiple approaches led to tree clusterization and the retrieval of possible target sequences that could be secreted into the extracellular environment. These strategies were useful not only for a whole-genome estimation of polyester-degrading gene products but also contributed to improving genome annotation of *Rhodococcus* strains.

The different behavior of the two strains in the growth and biodegradation of PCL suggests physiological differences and/or the use of slightly different enzymatic repertoires in the two strains. Indeed, RT-qPCR experiments on the selected genes showed that the lipase and protein mostly overexpressed by *R*. *erythropolis* D4 were different from those overexpressed by *R. opacus* R7.

However, it cannot be excluded that a more extensive, untargeted analysis will detect other genetic determinants of PCL degradation in the genomes of these *Rhodococcus* strains, expressed at a basal level or induced by polyester exposition.

Overall, our gene mining, although based on automatic annotation, improved our knowledge of the presence and organization of genes involved in polyester degradation in *R. erythropolis* D4 and *R. opacus* R7 genomes.

## 5 Conclusions

For the first time, this study demonstrates the biodegradative potential of bacteria belonging to the *Rhodococcus* genus and correlates these metabolic capabilities to their genomic traits by analyzing fully sequenced genomes and the genetic determinants expressed during the PCL biodegradation. Though the PCL biodegradative potential of *Rhodococcus* bacteria had already been identified, in-depth genomic and genetic determinant exploration of *Rhodococcus* members for PCL degradation had scarcely been undertaken. Indeed, we relied on a functional genomic approach with genome-based analyses, gene prediction, and clusterization, subsequently validated by gene expression analysis as a useful method for a full comprehension of how *Rhodococcus* strains exerted their degradative functions toward a biodegradable plastic such as PCL. Notwithstanding other molecular aspects that can be investigated, in the present study, we identified genomic and genetic traits useful for further biotechnological applications, including genetic engineering. Moreover, the results of our multidisciplinary approach lay the foundation for further approaches, i.e., transcriptomics and metabolomics approaches, and pave the way for biotechnological applications using *Rhodococcus* strains for the biodegradation of aliphatic polyesters.

## Data availability statement

The data presented in the study are deposited in the European Nucleotide Archive (ENA) repository, accession number PRJEB63643.

## Author contributions

JZ: Conceptualization, Data curation, Investigation, Methodology, Software, Validation, Visualization, Writing – original draft. DV: Data curation, Investigation, Methodology, Software, Writing – original draft, Visualization. SB: Funding acquisition, Project administration, Resources, Validation, Writing – review & editing. PD: Conceptualization, Project administration, Resources, Supervision, Writing – review & editing.

## Supplementary material

Supplementary Figure S1 Characterization of *Rhodococcus erythropolis* D4 for its identification. Morphological characteristics of *R. erythropolis* D4 on the LB agar plate (A) and *R. erythropolis* D4 cells after Gram staining under microscope (100X). Gel image of PCR product of 16S rRNA gene amplification (C); Line Mk, 500 to 10,000-bp ladder; D4, PCR product of 16S rRNA gene amplified from D4 genome; C-, negative control of oligonucleotides for 16S rRNA gene amplification.

Supplementary Figure S2 Chromatogram and list of the metabolic products deriving from PCL powder biodegradation by *Rhodococcus erythropolis* D4 and *Rhodococcus opacus* R7 obtained through GC-MSD analyses. The analyses were performed at the initial time **(A)**; after 28 and 42 days for D4 strain **(B, E)**; after 28 and 42 for R7 strain **(C, F)**, and after 28 and 42 days in the absence of inoculated cells (negative control) **(D, G)**.

Supplementary Figure S3 Clustering of *Rhodococcus erythropolis* D4 gene products (referred to as P) selected from those resembling nine reference sequences (referred to as R) through the two sequence filtering approaches. Numbers on the branches represent the bootstrap values calculated for the ML method from the package MEGA with 50 bootstraps. Abbreviations of protein names of *R. erythropolis* D4 are reported in Supplementary Table S2.

Supplementary Figure S4 Clustering of *Rhodococcus opacus* R7 gene products (referred to as P) selected from those resembling nine reference sequences (referred to as R) through the two sequence filtering approaches. Numbers on the branches represent the bootstrap values calculated for the ML method from the package MEGA with 50 bootstraps. Abbreviations of protein names of *R. opacus* R7 are reported in Supplementary Table S3.

Supplementary Table S1 List of oligonucleotides used for RT-qPCR analyses of *Rhodococcus erythropolis* D4 and *Rhodococcus opacus* R7 transcripts.

Supplementary Table S2 Gene products of *Rhodococcus erythropolis* D4 used for clustering analysis with reference proteins. The following features are included: protein names, ID from RAST, gene start and stop, aa length, and putative functions.

Supplementary Table S3 Gene products of *Rhodococcus opacus* R7 used for clustering analysis with reference proteins. The following features are included: protein names, ID from RAST, gene start and stop, aa length, and putative functions.

## References

[B1] Alcock B. P. Raphenya A. R. Lau T. T. Y. Tsang K. K. Bouchard M. Edalatmand A.. (2020). CARD 2020: antibiotic resistome surveillance with the comprehensive antibiotic resistance database. Nucleic Acids Res. 48, D517–D525. 10.1093/nar/gkz935 31665441 PMC7145624

[B2] Altschul S. F. Gish W. Miller W. Myers E. W. Lipman D. J. (1990). Basic local alignment search tool. J. Mol. Biol. 215, 403–410. 10.1016/S0022-2836(05)80360-2 2231712

[B3] Alvarez H. M. (2019). Biology of Rhodococcus. Cham: Springer Nature. 10.1007/978-3-030-11461-9

[B4] Andrews S. (2010). FastQC A Quality Control tool for High Throughput Sequence Data. Available online at: https://www.bioinformatics.babraham.ac.uk/projects/fastqc/ (accessed April 10, 2018).

[B5] Atanasova N. Paunova-Krasteva T. Kambourova M. Boyadzhieva I. (2023). A thermostable lipase isolated from *Brevibacillus thermoruber* strain 7 degrades ε-polycaprolactone. BioTech 12, 23. 10.3390/biotech12010023 36975313 PMC10046884

[B6] Auta A. S. Emenike C. U. Jayanthi B. Fauziah S. H. (2018). Growth kinetics and biodeterioration of polypropylene microplastics by *Bacillus* sp., *Rhodococcus* sp. isolated from mangrove sediment. Mar. Pollut. Bull. 127, 15–21. 10.1016/j.marpolbul.2017.11.036 29475646

[B7] Aziz R. K. Bartels D. Best A. A. DeJongh M. Disz T. Edwards R. A.. (2008). The RAST server: rapid annotations using subsystems technology. BMC Genomics 9, 75. 10.1186/1471-2164-9-75 18261238 PMC2265698

[B8] Bankevich A. Nurk S. Antipov D. Gurevich A. A. Dvorkin M. Kulikov A. S.. (2012). SPAdes: A new genome assembly algorithm and its applications to single-cell sequencing. J. Comput. Biol. 19, 455–477. 10.1089/cmb.2012.0021 22506599 PMC3342519

[B9] Bartnikowski M. Dargaville T. R. Ivanovski S. Hutmacher D. W. (2019). Degradation mechanisms of polycaprolactone in the context of chemistry, geometry and environment. Prog. Polym. Sci. 96, 1–20. 10.1016/j.progpolymsci.2019.05.004

[B10] Bolger A. M. Lohse M. Bjoern Usade B. (2014). Trimmomatic: a flexible trimmer for Illumina sequence data. Bioinformatics 3, 2114–2120. 10.1093/bioinformatics/btu170 24695404 PMC4103590

[B11] Bosi E. Donati B. Galardini M. Brunetti S. Sagot M.-F. Lió P.. (2015). MeDuSa: a multi-draft based scaffolder. Bioinformatics 31, 2443–2451. 10.1093/bioinformatics/btv171 25810435

[B12] Bradford M. M. (1976). A rapid and sensitive method for the quantitation of microgram quantities of protein utilizing the principle of protein-dye binding. Anal. Biochem. 72, 248–254. 10.1016/0003-2697(76)90527-3 942051

[B13] Budkum J. Thammasittirong S. N.-R. Thammasittirong A. (2022). High poly ε-caprolactone biodegradation activity by a new *Acinetobacter seifertii* isolate. Folia Microbiol. 67, 659–669. 10.1007/s12223-022-00964-7 35384558

[B14] Bustin S. A. Benes V. Garson J. A. Hellemans J. Huggett J. Kubista M.. (2009). The MIQE guidelines: minimum information for publication of quantitative real-time PCR experiments. Clin. Chem. 55, 611–622. 10.1373/clinchem.2008.112797 19246619

[B15] Chua T. K. Tseng M. Yang M.-K. (2013). (2013). Degradation of Poly(ε-caprolactone) by thermophilic *Streptomyces thermoviolaceus* subsp. *thermoviolaceus* 76T-2. AMB Express 3, 8. 10.1186/2191-0855-3-8 23360778 PMC3844369

[B16] Couvin D. Bernheim A. Toffano-Nioche C. Touchon M. Michalik J. Néron B.. (2018). CRISPRCasFinder, an update of CRISRFinder, includes a portable version, enhanced performance and integrates search for Cas proteins. Nucleic Acids Res. 46, W246–W251. 10.1093/nar/gky425 29790974 PMC6030898

[B17] Di Gennaro P. Rescalli E. Galli E. Guido S. Bestetti G. (2001). Characterization of *Rhodococcus opacus* R7, a strain able to degrade naphthalene and *o*-xylene isolated from polycyclic aromatic hydrocarbon-contaminated soil. Res. Microbiol. 152, 641–651. 10.1016/S0923-2508(01)01243-8 11605984

[B18] Di Gennaro P. Zampolli J. Presti I. Cappelletti M. D'Ursi P. Orro A.. (2014). Genome sequence of *Rhodococcus opacus* strain R7, a biodegrader of mono- and polycyclic aromatic hydrocarbons. Genome Announc. 2, e00827-14. 10.1128/genomeA.00827-14 25146139 PMC4153479

[B19] Gaby W. L. Hadley C. (1957). Practical laboratory test for the identification of *Pseudomonas aeruginosa*. J. Bacteriol. 74, 356–358. 10.1128/jb.74.3.356-358.1957 13475249 PMC314647

[B20] Grant J. R. Enns E. Marinier E. Mandal A. Herman E. K. Chen C.. (2023). Proksee: in-depth characterization and visualization of bacterial genomes. Nucleic Acids Res. 51, W484–W492. 10.1093/nar/gkad326 37140037 PMC10320063

[B21] Gricajeva A. Nadda A. K. Gudiukaite R. (2021). Insights into polyester plastic biodegradation by carboxyl ester hydrolases. J. Chem. Technol. Biotechnol. 97, 359–380. 10.1002/jctb.6745

[B22] Griebeler N. Polloni A. Remonatto D. Arbter F. Vardanega R. Cechet J. L.. (2011). Isolation and screening of lipase-producing fungi with hydrolytic activity. Food Bioprocess Technol. 4, 578–586. 10.1007/s11947-008-0176-5

[B23] Gupta N. Rathi P. Gupta R. (2002). Simplified para-nitrophenyl palmitate assay for lipases and esterases. Anal. Biochem. 311, 98–99. 10.1016/S0003-2697(02)00379-2 12441161

[B24] Gurevich A. Saveliev V. Vyahhi N. Tesler G. (2013). QUAST: quality assessment tool for genome assemblies. Bioinformatics 29, 1072–1075. 10.1093/bioinformatics/btt086 23422339 PMC3624806

[B25] Hakkarainen M. (2003). Qualitative and quantitative solid-phase microextraction gas chromatographic–mass spectrometric determination of the low molecular- mass compounds released from poly(vinyl chloride)/polycaprolactone–polycarbonate during ageing. J. Chromatogr. A 1010, 9–16. 10.1016/S0021-9673(03)01026-4 14503811

[B26] Hu X. Thumarat U. Zhang X. Tang M. Kawai F. (2010). Diversity of polyester-degrading bacteria in compost and molecular analysis of a thermoactive esterase from *Thermobifida alba* AHK119. Appl. Microbiol. Biotechnol. 87, 771–779. 10.1007/s00253-010-2555-x 20393707

[B27] Inglis G. D. Yanke L. J. Selinger L. B. (2011). Cutinolytic esterase activity of bacteria isolated from mixed-plant compost and characterization of a cutinase gene from *Pseudomonas pseudoalcaligenes*. Can. J. Microbiol. 57, 902–913. 10.1139/w11-083 22029433

[B28] Jain C. Rodriguez-R L. M. Phillippy A. M. Konstantinidis K. T. Aluru S. (2018). High throughput ANI analysis of 90K prokaryotic genomes reveals clear species boundaries. Nat. Commun. 9, 5114. 10.1038/s41467-018-07641-9 30504855 PMC6269478

[B29] Kanehisa M. Furumichi M. Sato Y. Kawashima M. Ishiguro-Watanabe M. (2023). KEGG for taxonomy-based analysis of pathways and genomes. Nucleic Acids Res. 51, D587–D592. 10.1093/nar/gkac963 36300620 PMC9825424

[B30] Khan I. Ray Dutta J. Ganesan R. (2017). *Lactobacillus* sps. lipase mediated poly (ε-caprolactone) degradation. Int. J. Biol. Macromol. 95, 126–131. 10.1016/j.ijbiomac.2016.11.040 27865950

[B31] Kobras C. M. Fenton A. K. Sheppard S. K. (2021). Next-generation microbiology: from comparative genomics to gene function. Genome Biol. 22, 123. 10.1186/s13059-021-02344-9 33926534 PMC8082670

[B32] Kosiorowsk K. E. Połomska X. Wang G. Borodina I. Mirończuk A. M. (2021). Efficient biodegradation of aliphatic polyester by genetically engineered strains of the yeast *Yarrowia lipolytica*. Int. Biodeterior. Biodegradation 161, 105232. 10.1016/j.ibiod.2021.105232

[B33] Kumar S. Stecher G. Li M. Knyaz C. Tamura K. (2018). MEGA X: molecular evolutionary genetics analysis across computing platforms. Mol. Biol. E 35, 1547–1549. 10.1093/molbev/msy096 29722887 PMC5967553

[B34] Lane D. J. (1991). “16S/23S rRNA sequencin g,” in Nucleic Acid Techniques in Bacterial Systematics, eds E. Stackebrandt, and M. Goodfellow (New York, NY: John Wiley and Sons), 115–175.

[B35] Li L. Lin X. Bao J. Xia H. Li F. (2022). Two Extracellular poly (ε-caprolactone)-degrading enzymes from *Pseudomonas hydrolytica* sp. DSWY01T: purification, characterization, and gene analysis. Front. Bioeng. Biotechnol. 10, 835847. 10.3389/fbioe.2022.835847 35372294 PMC8971842

[B36] Ma Q. Shi K. Su T. Wang Z. (2020). Biodegradation of polycaprolactone (PCL) with different molecular weights by *Candida antarctica* lipase. J. Polym. Environ. 28, 2947–2955. 10.1007/s10924-020-01826-4

[B37] Mandic M. Spasic J. Ponjavic M. Nikolic M. S. Cosovic V. R. O'Connor K. E.. (2019). Biodegradation of poly(ε-caprolactone) (PCL) and medium chain length polyhydroxyalkanoate (mcl-PHA) using whole cells and cell free protein preparations of *Pseudomonas* and *Streptomyces* strains grown on waste cooking oil. Polym. Degrad. Stab. 162, 160–168. 10.1016/j.polymdegradstab.2019.02.012

[B38] Maniatis T. Fritsch E. F. Sambrook J. (1982). Molecular Cloning: A Laboratory Manual. New York, NY: Cold Spring Harbor Laboratory, Cold Spring Harbor.

[B39] Masaki K. Kamini N. R. Ikeda H. Iefuji H. (2005). Cutinase-like enzyme from the yeast *Cryptococcus* sp. strain S-2 hydrolyzes polylactic acid and other biodegradable plastics. Appl. Environ. Microbiol. 71, 7548–7550. 10.1128/AEM.71.11.7548-7550.2005 16269800 PMC1287645

[B40] Mo A. Zhang Y. Gao W. Jiang J. He D. (2023). Environmental fate and impacts of biodegradable plastics in agricultural soil ecosystems. Appl. Soil. Ecol. 181, 104667. 10.1016/j.apsoil.2022.104667

[B41] Molitor R. Bollinger A. Kubicki S. Loeschcke A. Jaeger K. E. Thies S.. (2020). Agar plate-based screening methods for the identification of polyester hydrolysis by *Pseudomonas* species. Microb. Biotechnol. 13, 274–284. 10.1111/1751-7915.13418 31016871 PMC6922526

[B42] Murphy C. A. Cameron J. A. Huang S. J. Vinopal R. T. (1996). Fusarium polycaprolactone depolymerase is cutinase. Appl. Environ. Microbiol. 62, 456–460. 10.1128/aem.62.2.456-460.1996 8593048 PMC167813

[B43] Nawaz A. Hasan F. Shah A. A. (2014). Degradation of poly(ε-caprolactone) (PCL) by a newly isolated *Brevundimonas* sp. strain MRL-AN1 from soil. FEMS Microbiol. Lett. 362, 1–7. 10.1093/femsle/fnu004 25790487

[B44] Oh J. Y. Oh Y. R. Kim D. M. Eom G. T. Song J. K. (2022). Screening and efficient production of engineered lipase B from *Candida Antarctica* for eco-friendly recycling of waste polycaprolactone. Polym. Degrad. Stab. 195, 109807. 10.1016/j.polymdegradstab.2021.109807

[B45] Orro A. Cappelletti M. D'Ursi P. Milanesi L. Di Canito A. Zampolli J.. (2015). Genome and phenotype microarray analyses of *Rhodococcus* sp. BCP1 and *Rhodococcus opacus* R7: genetic determinants and metabolic abilities with environmental relevance. PloS ONE 10, e0139467. 10.1371/journal.pone.0139467 26426997 PMC4591350

[B46] Overbeek R. Begley T. Butler R. M. Choudhuri J. V. Chuang H. Y. Cohoon M.. (2005). The subsystems approach to genome annotation and its use in the project to annotate 1000 genomes. Nucleic Acids Res. 33, 5691–5702. 10.1093/nar/gki866 16214803 PMC1251668

[B47] Plastics Europe (ed.) (2022). Plastics - The Facts, 2022 ed. PlasticsEurope. Available online at: https://plasticseurope.org/knowledge-hub/plastics-the-facts-2022/ (accessed August 10, 2023).

[B48] Ribitsch D. Heumann S. Trotscha E. Acero H. E. Greimel K. Leber R.. (2011). Hydrolysis of polyethyleneterephthalate by *p*-nitrobenzylesterase from *Bacillus subtilis*. Biotechnol. Prog. 27, 951–960. 10.1002/btpr.610 21574267

[B49] Rosato A. Romano A. Totaro G. Celli A. Fava F. Zanaroli G.. (2022). Enzymatic degradation of the most common aliphatic bio-polyesters and evaluation of the mechanisms involved: an extended study. Polymers 14, 1850. 10.3390/polym14091850 35567020 PMC9101158

[B50] Seemann T. (2014). Prokka: rapid prokaryotic genome annotation. Bioinformatics 30, 2068–2069. 10.1093/bioinformatics/btu153 24642063

[B51] Shi K. Jing J. Song L. Su T. Wang Z. (2020). Enzymatic hydrolysis of polyester: degradation of poly(ε-caprolactone) by *Candida antarctica* lipase and *Fusarium solani* cutinase. Int. J. Biol. Macromol. 144, 183–189. 10.1016/j.ijbiomac.2019.12.105 31843602

[B52] Sievers F. Wilm A. Dineen D. Gibson T. J. Karplus K. Li W.. (2011). Fast, scalable generation of high-quality protein multiple sequence alignments using Clustal Omega. Mol. Syst. Biol. 7, 539. 10.1038/msb.2011.75 21988835 PMC3261699

[B53] Starikova E. V. Tikhonova P. O. Prianichnikov N. A. Rands C. M. Zdobnov E. M. Ilina E. N.. (2020). Phigaro: high-throughput prophage sequence annotation. Bioinformatics 36, 3882–3884. 10.1093/bioinformatics/btaa250 32311023

[B54] Strzelczyk P. Bujacz G. D. Kiełbasiński P. Błaszczyk J. (2015). Crystal and molecular structure of hexagonal form of lipase B from *Candida antarctica*. Acta Biochim. Pol. 63, 103–109. 10.18388/abp.2015_1065 26716135

[B55] Su X. Guo L. Ding L. Qu K. Shen C. (2016). Induction of viable but nonculturable state in *Rhodococcus* and transcriptome analysis using RNA-seq. PLoS ONE 11, e0147593. 10.1371/journal.pone.0147593 26808070 PMC4725852

[B56] Suzuki M. Tachibana Y. Kasuya K. (2021). Biodegradability of poly(3-hydroxyalkanoate) and poly(ε-caprolactone) via biological carbon cycles in marine environments. Polym. J. 53, 47–66. 10.1038/s41428-020-00396-5

[B57] Taboada B. Estrada K. Ciria R. Merino E. (2018). Operon-mapper: a web server for precise operon identification in bacterial and archaeal genomes. Bioinformatics 34, 4118–4120. 10.1093/bioinformatics/bty496 29931111 PMC6247939

[B58] Teufel F. Almagro Armenteros J. J. Johansen A. R. Gíslason M. H. Pihl S. I. Tsirigos K. D.. (2022). SignalP 6.0 predicts all five types of signal peptides using protein language models. Nat. Biotechnol. 40, 1023–1025. 10.1038/s41587-021-01156-3 34980915 PMC9287161

[B59] Thomas M. (1963). A blue peroxide slide catalase test. Mon. Bull. Min. Health 22, 124–125.

[B60] Tiago I. Teixeira I. Silva S. Chung P. Verissimo A. Manaia C. M. (2004). Metabolic and genetic diversity of mesophilic and thermophilic bacteria isolated from composted municipal sludge on poly-ε-caprolactones. Curr. Microbiol. 49, 407–414. 10.1007/s00284-004-4353-0 15696616

[B61] Tokiwa Y. Calabia B. P. Ugwu C. U. Aiba S. (2009). Biodegradability of plastics. Int. J. Mol. Sci. 10, 3722–3742. 10.3390/ijms10093722 19865515 PMC2769161

[B62] Tsuboi S. Yamamoto-Tamura K. Takada A. Yonemura S. Takada Hoshino Y. Kitamoto H.. (2022). Selection of p-nitrophenyl fatty acid substrate suitable for detecting changes in soil esterase activity associated with degradation of biodegradable polyester mulch films: a field trial. Ital. J. Agron. 17, 2040. 10.4081/ija.2022.2040

[B63] Urbanek A. K. Rymowicz W. Strzelecki M. C. Kociuba W. Franczak L. Mirończuk A. M.. (2017). Isolation and characterization of Arctic microorganisms decomposing bioplastics. AMB Expr. 7, 148. 10.1186/s13568-017-0448-4 28697585 PMC5503855

[B64] Winkler U. K. Stuckmann M. (1979). Glycogen, hyaluronate, and some other polysaccharides greatly enhance the formation of exolipase by *Serratia marcescens*. J. Bacteriol. 138, 663–670. 10.1128/jb.138.3.663-670.1979 222724 PMC218088

[B65] Won S.-J. Yim J. H. Kim H.-K. (2022). Functional production, characterization, and immobilization of a cold-adapted cutinase from Antarctic *Rhodococcus* sp. *Protein Expr. Purif* . 195–196, 106077. 10.1016/j.pep.2022.106077 35314296

[B66] Woodruff M. A. Hutmacher D. W. (2010). The return of a forgotten polymer-polycaprolactone in the 21st century. Prog. Polym. Sci. 35, 1217–1256. 10.1016/j.progpolymsci.2010.04.002

[B67] Yoshida S. Hiraga K. Takehana T. Taniguchi I. Yamaji H. Maeda Y.. (2016). A bacterium that degrades and assimilates poly(ethylene terephthalate). Science 351, 1196–1199. 10.1126/science.aad6359 26965627

[B68] Zampolli J. Collina E. Lasagni M. Di Gennaro P. (2014). Biodegradation of variable-chain-length *n*-alkanes in *Rhodococcus opacus* R7 and the involvement of an alkane hydroxylase system in the metabolism. AMB Express 4, 73. 10.1186/s13568-014-0073-4 25401074 PMC4230829

[B69] Zampolli J. Di Canito A. Cappelletti M. Collina E. Lasagni M. Di Gennaro P. (2020). Biodegradation of naphthenic acids: identification of *Rhodococcus opacus* R7 genes as molecular markers for environmental monitoring and their application in slurry microcosms. Appl. Microbiol. Biotechnol. 104, 2675–2689. 10.1007/s00253-020-10378-5 31993702

[B70] Zampolli J. Orro A. Manconi A. Ami D. Natalello A. Di Gennaro P. (2021). Transcriptomic analysis of *Rhodococcus opacus* R7 grown on polyethylene by RNA-seq. Sci. Rep. 11, 21311. 10.1038/s41598-021-00525-x 34716360 PMC8556283

[B71] Zampolli J. Orro A. Vezzini D. Di Gennaro P. (2022). Genome-based exploration of *Rhodococcus* species for plastic-degrading genetic determinants using bioinformatic analysis. Microorganisms 10, 1846. 10.3390/microorganisms10091846 36144448 PMC9506104

[B72] Zampolli J. Zeaiter Z. Di Canito A. Di Gennaro P. (2019). Genome analysis and -omics approaches provide new insights into the biodegradation potential of *Rhodococcus*. Appl. Microbiol. Biotechnol. 103, 1069–1080. 10.1007/s00253-018-9539-7 30554387

