# Supplementary figures and images for: Insights into the biodegradation of polycaprolactone through genomic analysis of two plastic-degrading Rhodococcus bacteria
Source: Front Microbiol. 2024 Jan 3;14:1284956. doi: 10.3389/fmicb.2023.1284956 (PMC10791956; doi:10.3389/fmicb.2023.1284956)

**Figure S1**

**A**

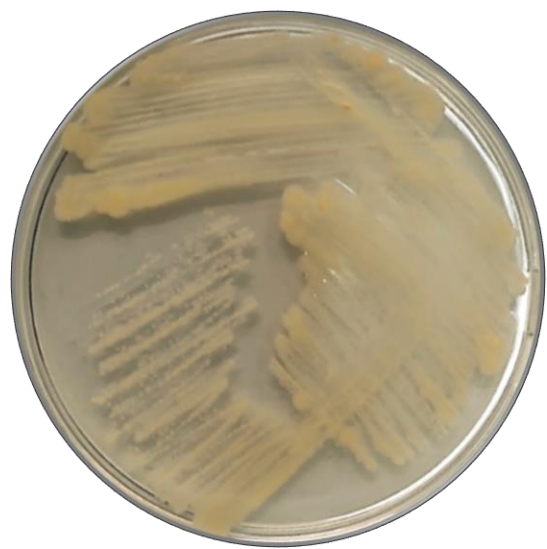

**B**

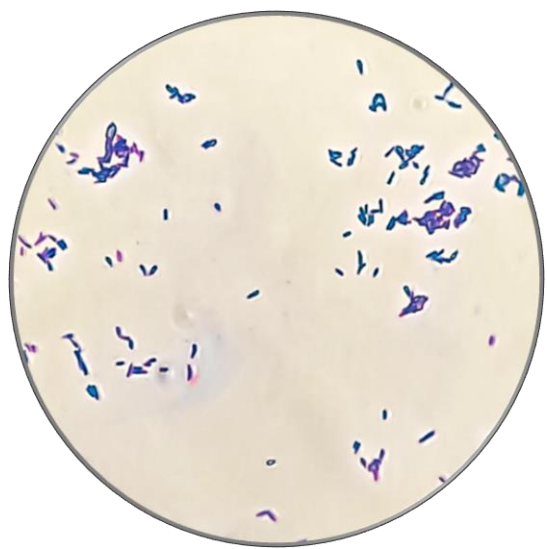

**C**

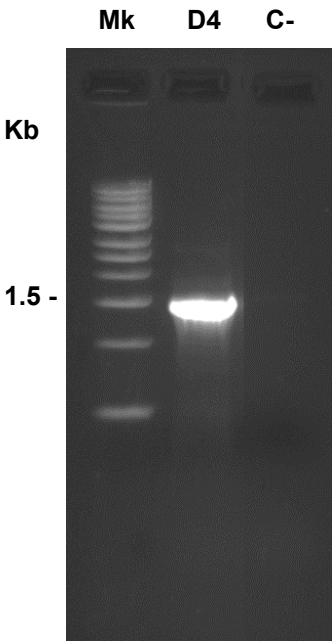

Supplement: Supplementary Figure S1 — Characterization of Rhodococcus erythropolis D4 for its identification. Morphological characteristics of R. erythropolis D4 on the LB agar plate (A) and R. erythropolis D4 cells after Gram staining under microscope (100X). Gel image of PCR product of 16S rRNA gene amplification (C); Line Mk, 500 to 10,000-bp ladder; D4, PCR product of 16S rRNA gene amplified from D4 genome; C-, negative control of oligonucleotides for 16S rRNA gene amplification. [file Data_Sheet_1.PDF]

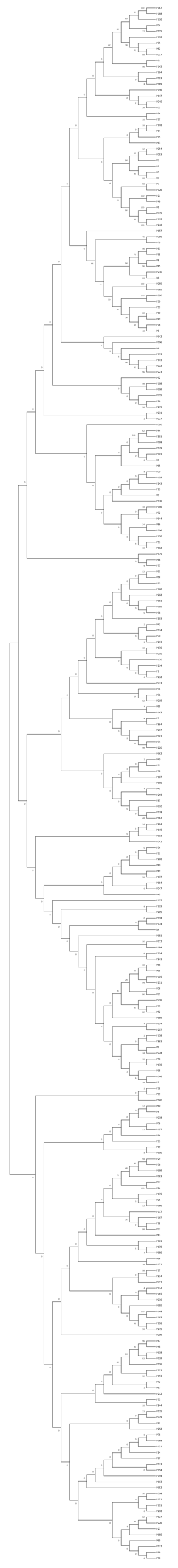

Supplement: Supplementary Figure S3 — Clustering of Rhodococcus erythropolis D4 gene products (referred to as P) selected from those resembling nine reference sequences (referred to as R) through the two sequence filtering approaches. Numbers on the branches represent the bootstrap values calculated for the ML method from the package MEGA with 50 bootstraps. Abbreviations of protein names of R. erythropolis D4 are reported in Supplementary Table S2. [file Data_Sheet_3.PDF]

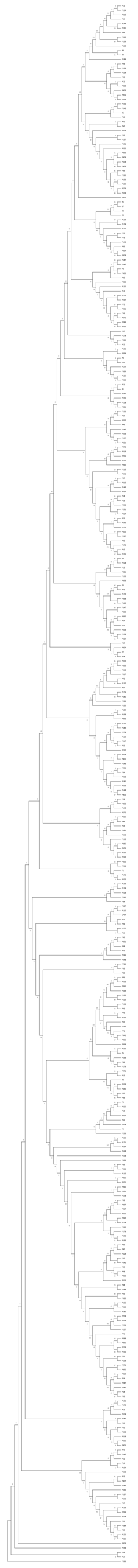

Supplement: Supplementary Figure S4 — Clustering of Rhodococcus opacus R7 gene products (referred to as P) selected from those resembling nine reference sequences (referred to as R) through the two sequence filtering approaches. Numbers on the branches represent the bootstrap values calculated for the ML method from the package MEGA with 50 bootstraps. Abbreviations of protein names of R. opacus R7 are reported in Supplementary Table S3. [file Data_Sheet_4.PDF]
