# Supplementary material for: Insights into the biodegradation of polycaprolactone through genomic analysis of two plastic-degrading Rhodococcus bacteria
Source: Front Microbiol. 2024 Jan 3;14:1284956. doi: 10.3389/fmicb.2023.1284956 (PMC10791956; doi:10.3389/fmicb.2023.1284956)

Figure S2

A

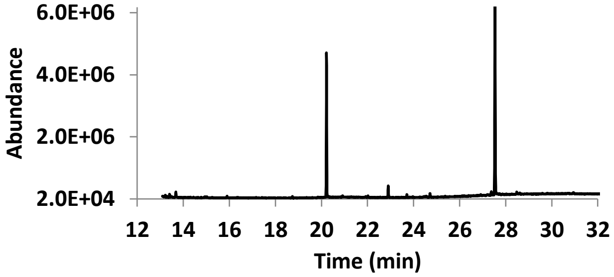

List of compound profile of PCL at initial time obtained through GC-MSD analyses after DCM extraction and derivatization.

Compound	Retention Time (t_R) (min)	Formula	CAS number
Cyclopentanecarboxylic acid, decyl ester	20.22	254	100028-04-1
Hexadecanoic acid	22.90	256	57-10-3
Octadecanoic acid	24.72	284	57-11-4
Glutaric acid ester derivative [a]	27.54	-	-

[a] Glutaric acid ester derivative, the mass spectra was similar only at 50% compared to NIST database.

B

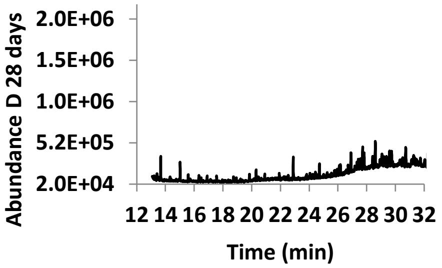

C

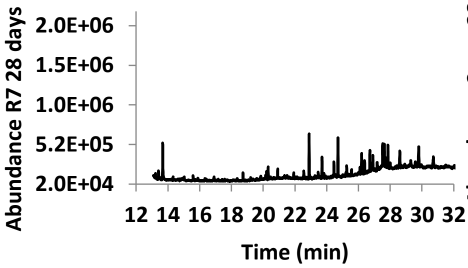

D

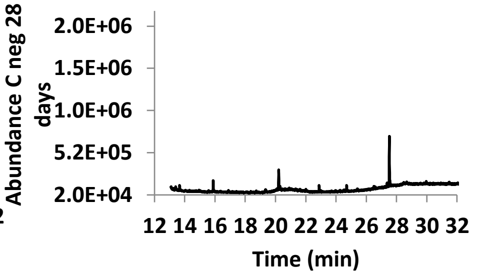

E

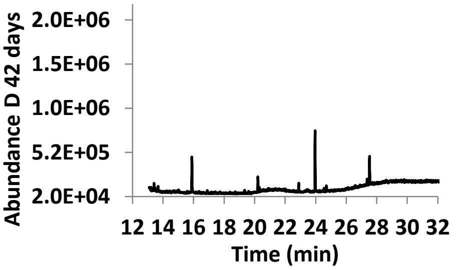

F

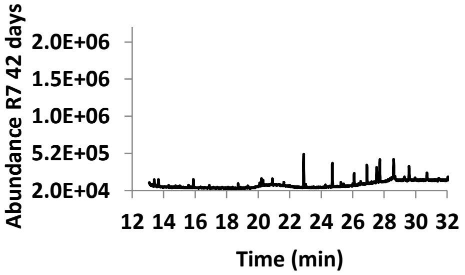

G

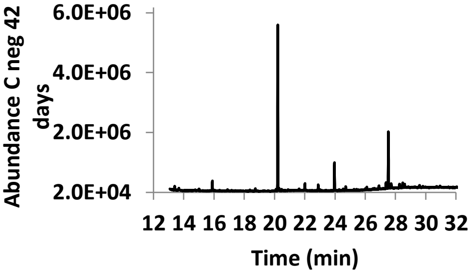

Supplement: Supplementary Figure S2 — Chromatogram and list of the metabolic products deriving from PCL powder biodegradation by Rhodococcus erythropolis D4 and Rhodococcus opacus R7 obtained through GC-MSD analyses. The analyses were performed at the initial time (A); after 28 and 42 days for D4 strain (B, E); after 28 and 42 for R7 strain (C, F), and after 28 and 42 days in the absence of inoculated cells (negative control) (D, G). [file Data_Sheet_2.PDF]
