# Supplementary material for: Insights into the biodegradation of polycaprolactone through genomic analysis of two plastic-degrading Rhodococcus bacteria
Source: Front Microbiol. 2024 Jan 3;14:1284956. doi: 10.3389/fmicb.2023.1284956 (PMC10791956; doi:10.3389/fmicb.2023.1284956)
Supplement: Supplementary Table S1 — List of oligonucleotides used for RT-qPCR analyses of Rhodococcus erythropolis D4 and Rhodococcus opacus R7 transcripts. [file Table_1.DOCX]

**Table S1.** List of oligonucleotides used for RT-qPCR analyses.

| **Oligonucleotide name** | **sequence (5’ - 3’)** |
| --- | --- |
| *R. erythropolis* D |  |
| RT-D16S-f | AACCTCTTTCAGCAGGGAC |
| RT-D16S-r | ACAAACGACGCGACAAAC |
| RT-D-P152-f | AAGCAACTTCGCCATCAC |
| RT-D-P152-r | ACCGGCAAGATAGAGAACC |
| RT-D-P3109-f | CATCGCACACGAGACTTAC |
| RT-D-P3109-r | GAACCAACCCGAACATCAC |
| *R. opacus* R7 |  |
| RT-16S-R7f | TCGTGAGATGTTGGGTTAAG |
| RT-16S-R7r | CCTCTGTACCGGCCATTGTAG |
| RT-R7-P7703-F | TACTGCAGCTCGCGATCAA |
| RT-R7-P7703-R | TCCGACACCTTGACCAGAAC |
